# A High Resolution Genome-Wide Scan of HNF4α Recognition Sites Infers a Regulatory Gene Network in Colon Cancer

**DOI:** 10.1371/journal.pone.0021667

**Published:** 2011-07-28

**Authors:** Fridtjof Weltmeier, Juergen Borlak

**Affiliations:** 1 Department of Molecular Medicine and Medical Biotechnology, Fraunhofer Institute of Toxicology and Experimental Medicine, Hannover, Germany; 2 Centre for Pharmacology and Toxicology, Hannover Medical School, Hannover, Germany; University of Georgia, United States of America

## Abstract

The hepatic nuclear factor HNF4α is a versatile transcription factor and controls expression of many genes in development, metabolism and disease. To delineate its regulatory gene network in colon cancer and to define novel gene targets a comprehensive genome-wide scan was carried out at a resolution of 35 bp with chromatin IP DNA obtained from the human colon carcinoma cell line Caco-2 that is a particularly rich source of HNF4α. More than 90% of HNF4α binding sites were mapped as promoter distal sequences while enhancer elements could be defined to foster chromatin loops for interaction with other promoter-bound transcription factors. Sequence motif analysis by various genetic algorithms evidenced a unique enhanceosome that consisted of the nuclear proteins ERα, AP1, GATA and HNF1α as cooperating transcription factors. Overall >17,500 DNA binding sites were identified with a gene/binding site ratio that differed >6-fold between chromosomes and clustered in distinct chromosomal regions amongst >6600 genes targeted by HNF4α. Evidence is presented for nuclear receptor cross-talk of HNF4α and estrogen receptor α that is recapitulated at the sequence level. Remarkably, the Y-chromosome is devoid of HNF4α binding sites. The functional importance of enrichment sites was confirmed in genome-wide gene expression studies at varying HNF4α protein levels. Taken collectively, a genome-wide scan of HNF4α binding sites is reported to better understand basic mechanisms of transcriptional control of HNF4α targeted genes. Novel promoter distal binding sites are identified which form an enhanceosome thereby facilitating RNA processing events.

## Introduction

Hepatic nuclear factor HNF4α is a member of the nuclear receptor superfamily and an extremely versatile transcription factor [1]. This zinc finger protein is expressed in liver, intestine, pancreas and other tissues, and binds to cognate DNA sequences as a homodimer [2]. In the past, some dozen promoter binding sites were reported. The use of chromatin immunoprecipitation and microarray hybridization ChIP-chip methodologies demonstrated that these are only the smallest fraction of the actual HNF4α binding sites. By use of tiling array encompassing the ENCODE regions that represent 1% of the genome in the human hepatoma cell line HepG2 [3] a total of 194 HNF4α binding sites could be mapped. In another study HNF4α binding sites in hepatocytes and pancreatic islets were mapped, but the approach focused on promoter regions only [4]. As of today, a genome-wide footprint of HNF4α has not been reported. Notably, HNF4α is a master regulatory protein and dysfunction of HNF4α has been associated with metabolic and cancerous diseases. We were particularly interested in exploring an HNF4α genomic footprint in the human colon adenocarcinmoa Caco-2 cell line that has been widely used to explore HNF4α activity [5] thereby identifying a network of regulated genes. Specifically, the cell line differentiates into enterocytes upon confluence [6] and expresses HNF4α protein comparable to liver [7]. Here we report the first genome-wide scan that enabled an identification of >17,500 binding sites targeted by HNF4α and describe their chromosomal distribution. Additionally, we studied the consequences of HNF4α protein induction on transcriptional activity of *de novo* identified genes and demonstrate good agreement between novel gene targets and their expression in Caco-2 cells. Finally, we analyzed HNF4α binding sites for enriched binding motifs and identified cooperating transcription factors that appeared to act in concert with HNF4α in an enhanceosome of transcriptional regulation.

## Results

**Figure 1:**
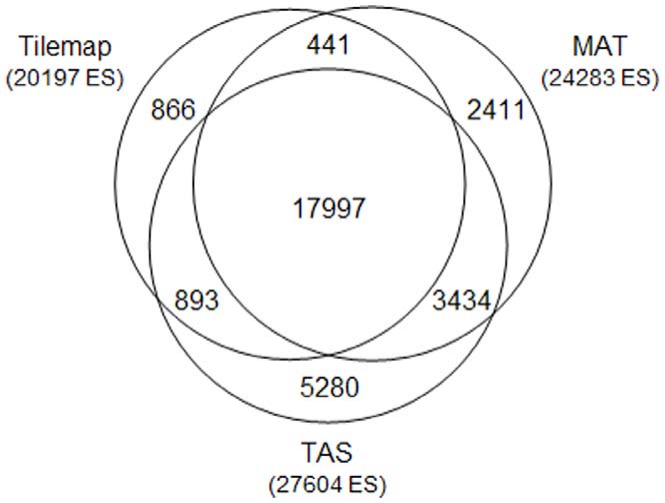
Venn diagram of overlap between HNF 4α binding sites identified by different algorithms. Raw data was analyzed with three different programs (Tilemap, MAT and TAS) to identify HNF4α binding sites. Although different parameter settings (e.g. band width of 200, 300 and 400 nucleotides) and different algorithms were used, the overlap was surprisingly high. The Venn diagram was calculated using the intersect function of Galaxy [47].

Chromatin IP experiments were performed with Caco-2 cell cultures and an antibody highly specific for HNF4α. Notably, total input as well as IP-DNA from three independent biological replicates was obtained and subjected to an optimized protocol for unbiased amplification according to the manufactures recommendation (see also Material and Method section). The amplified DNA from independent experiments was hybridized to Affymetrix Human tiling 2.0R arrays with a genome-wide resolution of 35 bp. Then, raw data were examined for enriched regions by use of three independent algorithms (TAS [8], MAT [9] and Tilemap [10]). Initial cutoff criteria were set on the weakly enriched positive control (*OTC*) and further improved based on the frequency of HNF4α -motifs within the enriched regions, as determined by the MATCH algorithm [11]. To gain confidence in the data, results from the three algorithms were intersected. The overlap of enrichment sites (ES) identified by the three approaches was very high (**Fig. 1**), even though small differences were observed possibly due to the different repeat libraries used. Overall this approach led to an identification of 17,561 ES (**Table S1**). Moreover, a low stringency data set was generated by merging ES data detected with the MAT and Tilemap algorithms. This resulted in a total of 25,419 ES (**Table S2**).

**Figure 2:**
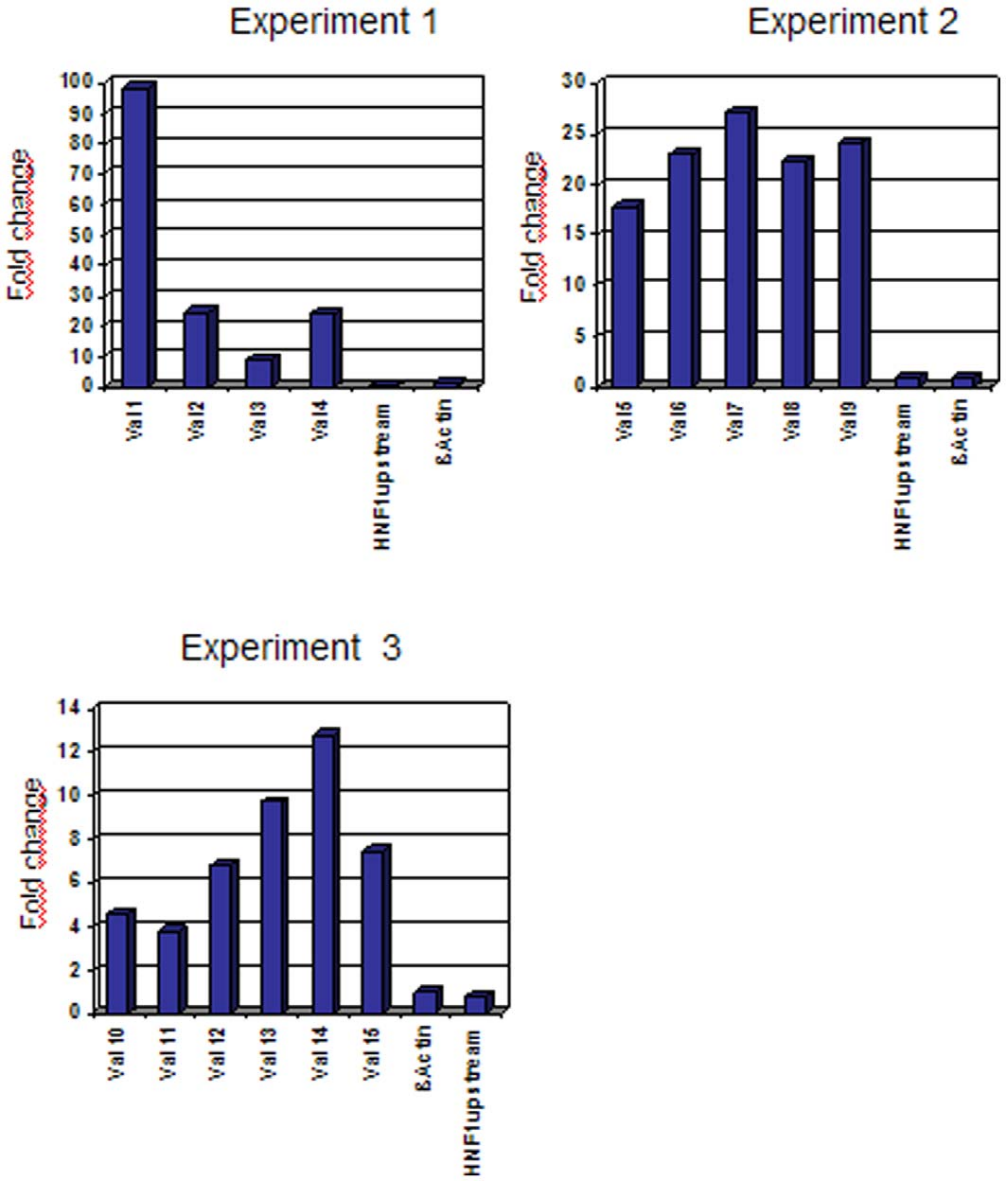
Validation of HNF4α binding sites. Enrichment of novel HNF4α binding sites detected by ChIP-chip was confirmed by real time PCR. ChIP-DNA from three independent experiments was used. Normalization was performed using a β-actin negative control, and the values are shown as fold enrichment versus total input. HNF1α upstream is a second negative control, located upstream of the known HNF4α binding site in the HNF1α promoter and is used to confirm the ß-actin negative control.

Additionally, 15 ES of known HNF4α gene targets were chosen and their enrichment in the primary IP-DNA was determined by realtime quantitative PCR. For all selected sites enrichment could be confirmed. Thus the robustness and quality of the data was validated (**Fig. 2**). Among the identified ES there were HNF4α binding sites already described in the literature or reported elsewhere such as *AAT* (R00114), *GCC* (R08885), *PCK* (R12074), *APOB* (R01612), *CYP2C9* (R15905), *AKR1C4* (R13037), *ACADM* (R15923) or *CYP27A1* (R15917). In the case of SHBG (R15941), ES were determined within a few hundred base pairs relative to the reported binding sites. Other binding sites described in literature, like *ALDH2* (R15845), could not be confirmed. However, quantification by real time PCR showed that the *ALDH2* site was not enriched in the primary IP-DNA. As the HNF4α protein functions in a tissue specific manner, it is not unexpected that some ES are not bound in Caco-2 cells; their accessibility rather depends on chromatin organization, which in turn depends on the cell type. This is supported by independent investigations, where significant differences in DNA binding sites in different cell types had been observed [12].

### The HNF4α motif is highly enriched within the ChIP regions

ChIP-enriched regions were examined for HNF4α binding motifs with the MATCH algorithm [11]. Using stringent criteria to minimize false positives, >14-fold enrichment was observed for HNF4α targeted sequences when compared to genomic background (**Table S3**).

**Figure 3:**
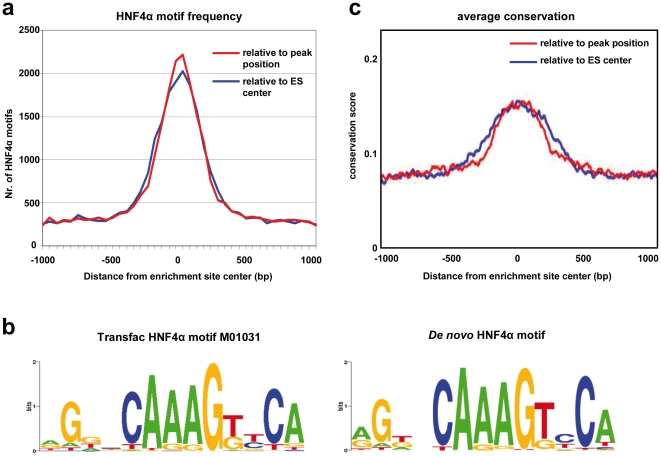
Enriched regions contain HNF4α binding motifs that are highly conserved. a) HNF4α motifs in the area of 1,000 bp surrounding enrichment site (ES) peak or center positions as detected with MATCH, using cutoffs to minimize false positives. The distance of the center of detected motifs to the peak or center position of the ES was calculated. A histogram was created using bins of 50 nucleotides around the center or peak positions. The blue line shows the deviation of HNF4α motifs relative to the ES center, the red line shows the deviation relative to the peak position. b) HNF4α ChIP-chip ES to allow easy *de novo* prediction of the HNF4α binding motif. After analysis of the sequence of regions enriched by HNF4α ChIP-chip with Gibbs motif sampler, the HNF4α-motif was actually detected two times, with the second motif presenting only half a site. c) Conservation of all HNF4α binding sites (blue line). ES centers (blue) or Peak positions (red) were extended to 1,000 bp in both directions, and for each nucleotide the average conservation score, based on the high-quality phast-Cons information from the UCSC GoldenPath Genome Resource, was calculated. The average conservation scores were plotted against the nucleotides position. Analyses were performed with CEAS [43].

The regions of 500 bp surrounding the 17,561 identified binding sites were analyzed for HNF4α motifs with settings to minimize false negatives by use of the MATCH algorithm [11]. Essentially, regions were segmented into bins of 25 bp, and the number of occurrences of the different motifs within each bin was counted. This resulted in a total of 23,145 motifs and equates to 1.32 motifs / ChIP region. For 98.1% of the ChIP regions at least one motif was detected. This suggests that most of the ChIP regions were enriched due to direct binding of HNF4α. By the same approach the binding sites reported for the ENCODE regions [3] were examined and 1.13 motifs / Chip region were estimated which is less than observed in the present study to possibly suggest high resolution tiling arrays to better identify ES. Subsequently, the distribution of the HNF4α motifs around the center of the ChIP enriched regions was analyzed (**Fig. 3a**). The majority of motifs is located in a region of only ∼500 base pairs. When the enriched regions at the peak positions detected with the MAT algorithm were aligned against the center position, the number of HNF4α motifs increased, therefore indicating that the peak position better estimates actual binding site. Additionally, the Gibbs motif sampler was applied to identify ES regions to enable easy *de novo* definition of the HNF4α motif (**Fig. 3b**).

To underline the biological importance of the identified binding sites their average conservation was studied as well. The nucleotides in the center, where a binding site could be expected, show a two times higher conservation than those at the ends of the plot (genomic background) (**Fig. 3c**). Again, when the ChIP enriched regions were aligned by the peak position, the conservation peak was even better defined.

### HNF4α binds predominantly to enhancer elements

**Figure 4:**
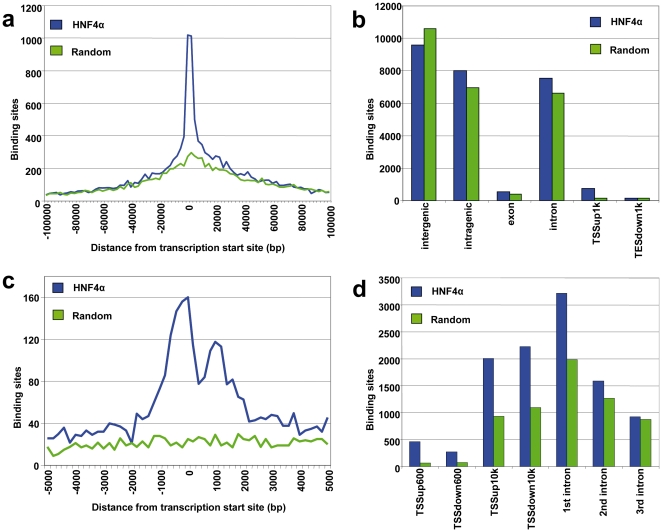
Distribution of ES relative to RefSeq loci. a) Location of HNF4α binding sites relative to the closest TSS of RefSeq genes as compared to random distribution. The regions of 100,000 nucleotides surrounding each TSS were divided into bins of 5,000 nucleotides, and the number of binding sites in each bin was counted. b) Genomic distribution of HNF4α binding sites. The number of binding sites located in the specified regions of RefSeq annotated genes was calculated by the software tool CisGenome. TSSup1k: 1,000 bp upstream of a transcription start site (TSS); TESdown1k: 1,000 bp downstream of a transcription end site. c) Distribution of HNF4α binding sites located proximal to TSS of RefSeq genes, compared to random distribution. The regions of 5,000 nucleotides surrounding each TSS were divided into bins of 200 nucleotides, and the number of binding sites in each bin was counted. d) Overrepresentation of HNF4α binding sites in upstream and downstream TSS proximal regions and in first, second an third introns of RefSeq annotated genes, relative to a random control regions. The positions of TSS, first, second and third introns of RefSeq annotated genes were retrieved from UCSC, and the number of HNF4α binding sites located in the specified regions was calculated. TSSup600: 600 bp upstream of a transcription start site; TSSdown600: 600 bp downstream of a transcription start site; TSSup10k: 10,000 bp upstream of a TSS; TSSdown10k: 10,000 bp downstream of a TSS.

The distance from HNF4α binding site to the closest transcription start site (TSS) of a RefSeq gene was determined. Here, a nearly 5-fold overrepresentation of binding sites in the promoter region of −1000 to 0 relative to the TSS was observed (**Fig. 4a, b**). However, only 5,8% of all binding sites mapped to promoter-proximal regions and 3,6% of all RefSeq promoters are bound by HNF4α. A similar and significant lack of preference for binding to 5′ promoter-proximal regions had been reported for the transcription factors Sp1, P53, cMyc and ERα [13], [8]. While some transcription factors like E2F1 show a clear preference for 5′ promoter-proximal regions [14], accumulating evidence is highly suggestive for promoter-proximal regions to constitute only a small fraction of mammalian gene regulatory sequences. Indeed, some of the nuclear receptors display higher activity at enhancer rather than promoter binding sites [13], [15]. Consequently, studies with promoter arrays are of limited value.

An analysis of the distribution of ES at 600 bp surrounding the TSS provided evidence for preferential binding in the upstream region (**Fig. 4c and d**). However, at a distance greater than 800 bp of the TSS, more binding sites are located downstream. Notably, many transcription factors binding sites are located in the first intron; the second peak shown in **Fig. 4c** is due to binding of intronic regions. The frequency of HNF4α binding sites in RefSeq annotated genes was further analyzed. This evidenced an overrepresentation of ES in the first introns, but less so in the second or third (**Fig. 4d**).

**Figure 5:**
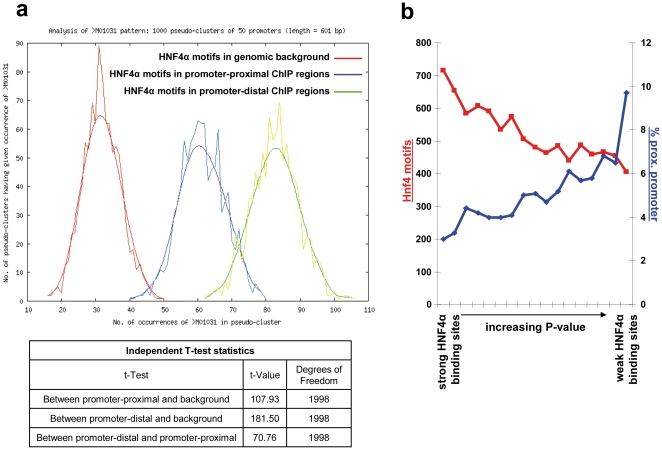
Promoter-proximal ES of HNF4α. a) Bootstrapping analysis of HNF4α binding motif (matrix M01031) in promoter-proximal and promoter distal regions. 100 promoter-proximal ES (−138 to −2 relative to the TSS) were compared to 100 promoter-distal ES (−24972 to −23489 relative to the TSS) by the bootstrapping analysis tool POBO [48]. Promoter-proximal ES show a significantly lower number of HNF4α motifs. b) ES (300 bp surround the peak position) were sorted by their P-value (as calculated by the MAT algorithm) and divided into bins of 1,000 ES. For each bin the number of HNF4α motif occurrences and the percentage of promoter-proximal ES were calculated. As can be seen, ES with a high P-value (weak ES) are more likely to be located promoter-proximal but to contain no HNF4α binding motif.

Importantly, a recent HNF4α ChIP-chip study suggested promoter-proximal ES are due to indirect interactions of HNF4α with other transcription factors [3]. Consequently, a model was developed whereby HNF4α binds to distant enhancer elements and creates chromatin loops by interacting with other promoter-bound transcription factors. Unfortunately, this model was based on less than 1% of genomic sequences. Based on the genome wide scan reported herein HNF4α binding motifs in promoter-distal regions are overrepresented as compared to promoter-proximal regions (**Fig. 5a**), nonetheless regions with low enrichment display a higher percentage of promoter-proximal binding sites than regions with high enrichment (**Fig. 5b**). Possibly, HNF4α contacts promoter-proximal regions by physical interaction with other transcription factors and therefore displays promoter as well as an enhancer binding activity.

**Figure 6:**
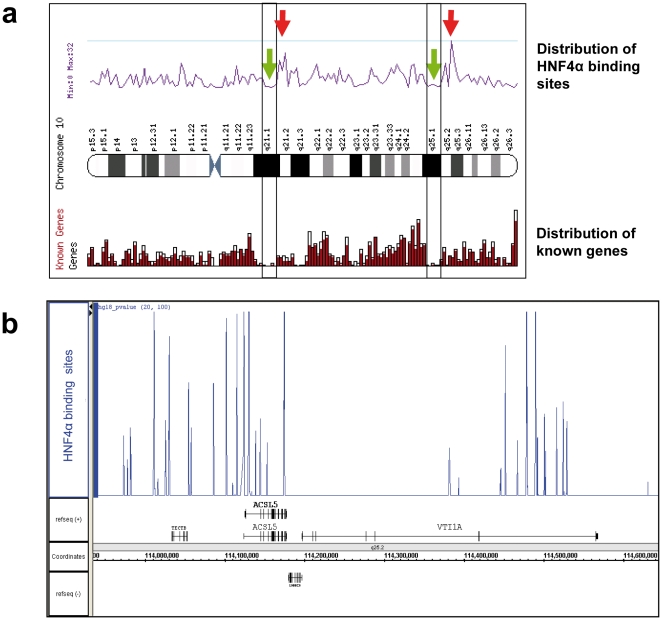
Cluster of HNF4α binding sites. a) The distribution of HNF4α binding sites (purple line chart, upper half) is compared to the distribution of known genes on chromosome 10. Green arrows mark two gene-sparse regions in which ES are found. The red arrows mark two regions with a high number of HNF4α binding sites and a low number of genes. Analyses were performed using the Ensembl tool Karyoview (http://www.ensembl.org/Homo_sapiens/karyoview). b) Clusters of HNF4α binding sites in a genomic region on chromosome 10 with high content of binding sites (the region marked by the second red arrow in a)). The binding sites identified in this study, displayed as blue peaks in the upper half, are presented using the IGB genome browser. The binding sites are distributed in two clusters around the transcription start site of the ACSL5 locus and in the 3′-region of the VTI1A locus.

**Figure 7:**
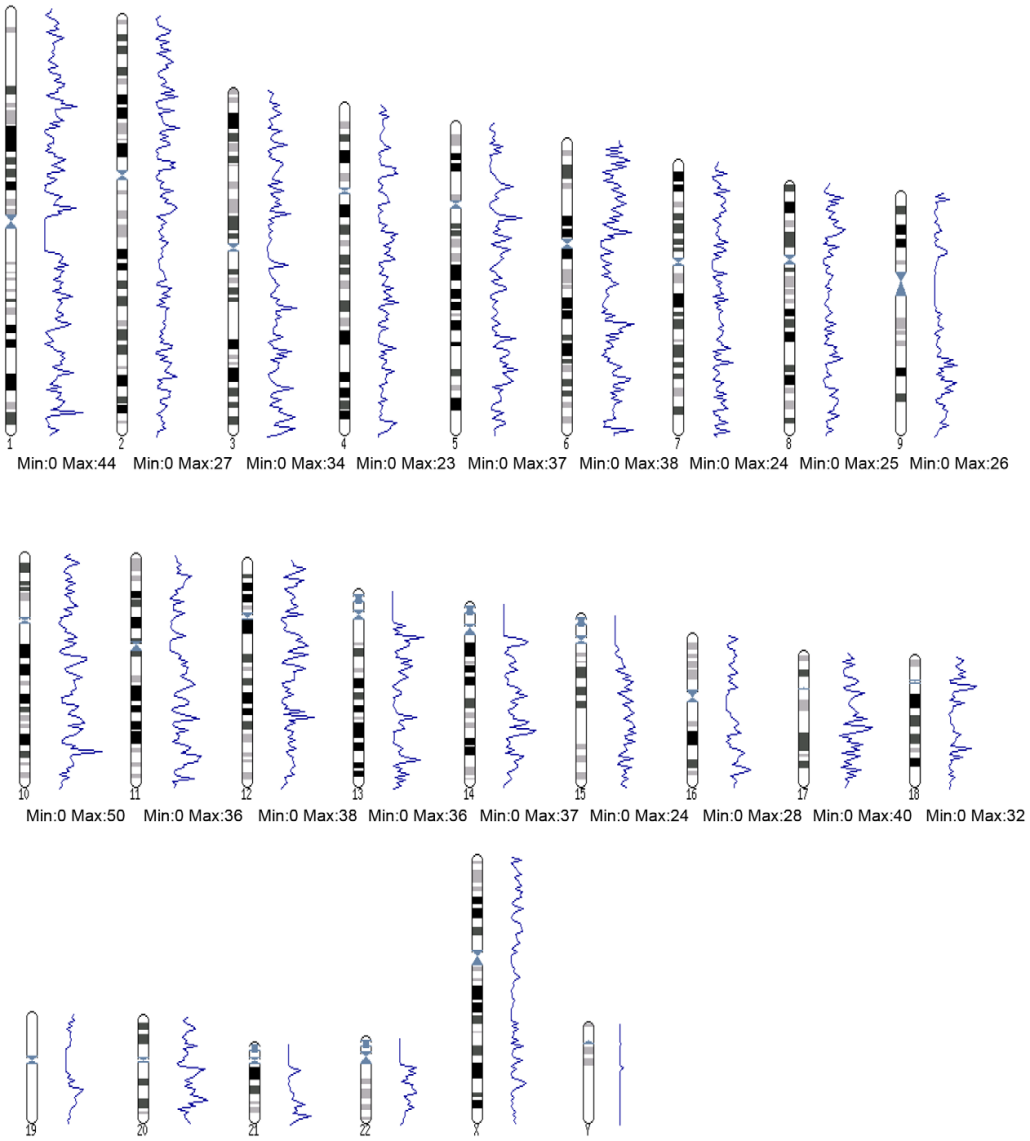
Chromosomal distribution of HNF4α ChIP enrichment sites. Each chromosome was divided into 150 ‘*bins*’, and within each bin the number of ES was counted. In the blue line chart, the number of HNF4α binding sites within each bin is represented as a single data point. Below each chromosome the minimum and maximum number of binding sites located in a single bin is given. Analyses were performed using the Ensembl tool Karyoview (http://www.ensembl.org/Homo_sapiens/karyoview).

The distribution of identified ES across the chromosomes varied >6 fold. Strikingly, the Y chromosome is devoid of HNF4α ES (**Table S6**) and the chromosomal distribution of ES is not randomly distributed; rather clusters are formed (**Fig. 6a** **;** **Fig. 7**). These clusters are not related to differences in the gene density within these regions, as shown for chromosome 10. The region with the highest density of binding sites on chromosome 10 contains two clusters of binding sites with the overlapping loci ACSL5 and VTI1A1 (**Fig. 6b**). By scanning the genomic sequence for windows of 100,000 bp that contain ≥10 HNF4α binding sites, fifteen clusters could be defined (**Table S7**). Indeed, most enhancers appear to be promiscuous and thus regulate multiple genes [16]. While enhancer activity may take place over hundreds of kilobases [17] and even cases of inter-chromosomal regulation by enhancers have been reported [18], most are within 100,000 bp of their respective TSS. To better define a possible enhanceosome for target genes sequences closest to RefSeq genes with a TSS separated by less than 100,000 nucleotides were selected (**Table S8**).

### HNF4α transcription factor cross-talk

**Figure 8:**
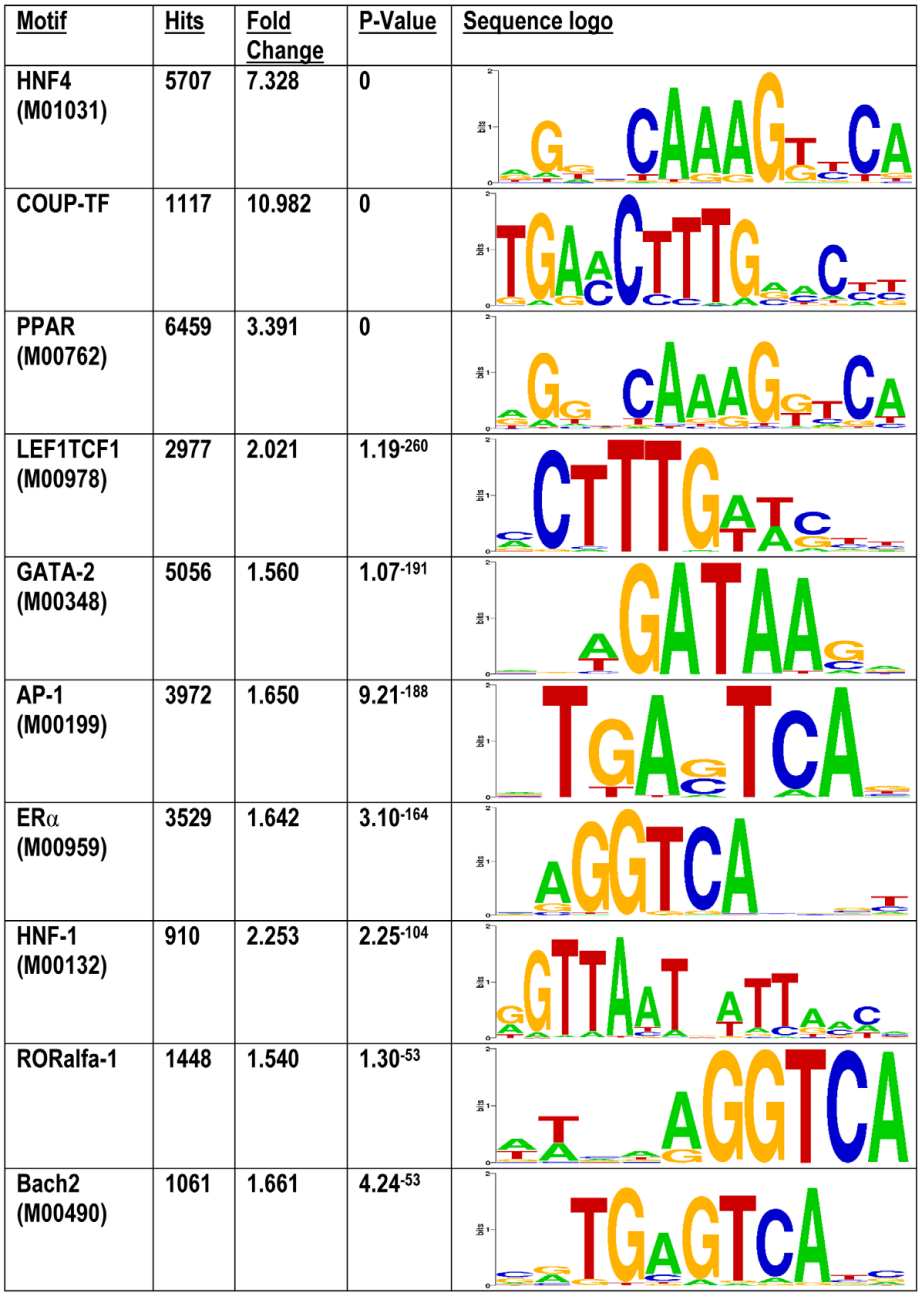
Motifs overrepresented within the ChIP-enriched regions. The 10,000 most significant ChIP-enriched regions were analyzed for overrepresented motifs by use of the CEAS tool [43]. Shown are the 10 motifs with the most significant enrichment, while redundant motifs (i.e. multiple motifs for the same transcription factor) were removed. The similarity or dissimilarity of the motifs is visualized by using Weblogo depiction (http://weblogo.berkeley.edu/). Motif enrichment analysis with Genomatix RegionMiner and MATCH [11] are found in **Tables S3, S4, S5**.

**Figure 9:**
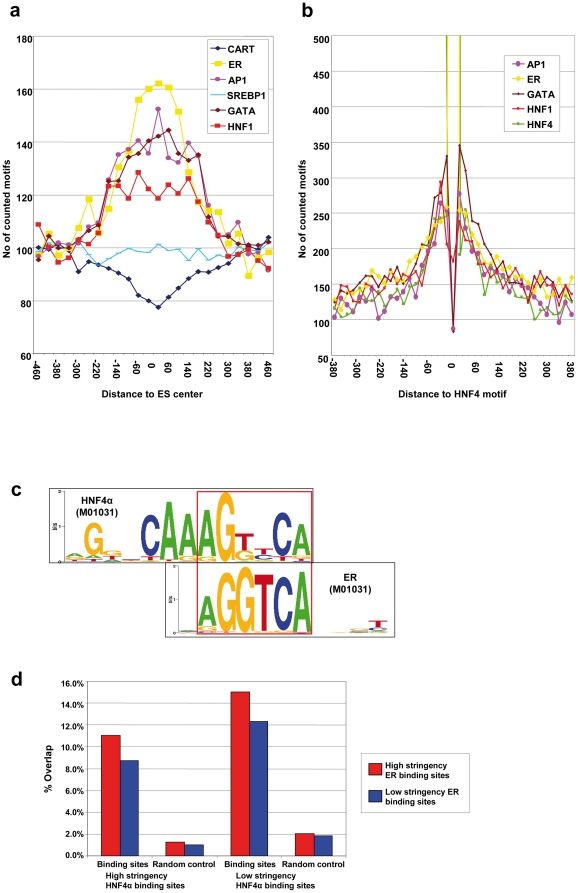
Distribution of AP1, CART, ERα, GATA2, HNF1α and SREBP motifs within regions enriched by HNF4α-ChIP. a) Peak positions (represented as 0) were extended to 500 bp in both directions, and Motifs were detected by use of the MATCH algorithm [11] using cutoff criteria to minimize the sum of false positives and false negatives. Regions were segmented into bins of 25 bp, and the number of occurrences of the different motifs within each bin was counted. b) Plot of the relative distance of HNF4α motifs to other motifs enriched in the ChIP region. Within ChIP regions the most conserved HNF4α motifs where identified. The sequences of the 500 nucleotides surrounding these most conserved HNF4α motifs where retrieved and analyzed for those motifs of other TF that were also enriched in the ChIP regions. Then, the distance between these motifs and the HNF4α motif was calculated using CisGenome for motif detection and plotted as histogram using bins of 20 bp. The HNF4α motif is found at the center, reaching from bp −6 to bp +6. **HNF4α and ERα share common and overlapping binding sites.** c) Display of the overlap between the binding motifs of HNF4α and the estrogen receptor (ERα) by use of Weblogo illustrations. Both motifs show a partial overlap. d) Overlap between ERα binding sites and HNF4α binding sites. The high stringency set of ERα binding sites identified by ChIP-chip [13] was obtained. The percentage of ERα binding sites identified in this study and also bound by HNF4α is displayed in a bar chart. The overlap of ERα binding sites with random control regions was determined.

To search for transcription factor cross-talk the ChIP regions for overrepresented motifs were considered. Among the motifs with the highest enrichment are matrices similar to the HNF4α binding motif, e.g. those for COUP-TF, PPAR or LEF1 (**Fig. 8** **, Tables S3, S4, S5**). These transcription factors are known to compete with HNF4α for common binding sites [19]–[21]. However, many motifs dissimilar to HNF4α, e.g. the binding motifs for HNF1α, AP1 or GATA transcription factors, were also significantly enriched. If these factors act in common with HNF4α, it could be expected that the frequency of their motifs increases with decreasing distance to the HNF4α binding sites. Therefore, the frequency of such motifs relative to the HNF4α binding sites was analyzed (**Fig. 9a and 9b**). The enrichment of these motifs is restricted to a region of a few hundred base pairs around the peak position, therefore supporting the idea that they are part of an enhanceosome defined by HNF4α. Besides an increase in the frequency of binding motifs for AP1, GATA, ERα and HNF1α an inverse relationship between HNF4α and CART motifs was observed, but there was no relationship with SREBP1 (**Fig. 9a**). It is tempting to speculate that this is of regulatory importance for HNF4α. Other analyzed motifs showed only a slight correlation between the number of motifs and the distance to the peak position, although they were clearly enriched in ChIP regions (e.g. USF, CREB, HNF6).

The high sequence similarity of binding sites for HNF4α and estrogen receptor (ERα) is of considerable importance (**Fig. 9c**). To further analyze the probability of co-occupancy of enriched motifs the HNF4α binding sites were determined exactly by motif analysis. The genomic position of the highest scoring HNF4α motif within the ChIP regions was retrieved and extended to 500 nucleotides to the left and right flanking sequences. Within these sequences, other enriched motifs were detected and the distance to the HNF4α motif was calculated (**Fig. 9b**). As expected, most ERα motifs co-locate at HNF4α ES causing a high peak at the center. In contrast, HNF1α, AP1 and GATA motifs display enrichment at a distance of 20 to 60 nucleotides to the HNF4α motif. There is also an enrichment of less conserved HNF4α binding sites in close proximity to the highest scoring HNF4α motif. This overrepresentation of less conserved HNF4α motifs may play a role in increasing the probability of HNF4α binding at the local sequence context surrounding the binding site.

As the ERα motif overlaps partially with the HNF4α motif, it is tempting to speculate that such enrichment within the ChIP regions is due to a functional connection between the two factors. Recently, a genome-wide map of ERα binding sites was reported [13]. Therefore the data for ERα and HNF4α sites were analyzed and found to considerably overlap (**Fig. 9d**). Using either the low or high stringency set of HNF4α or ERα binding sites up to about 15% of the ERα binding sites were also targeted by HNF4α, thus supporting the idea of cooperation between HNF4α and the ERα nuclear receptor. Importantly, several independent investigations report synergism in the transcription factor activity of HNF4α and ERα in the gene regulation of, for instance, apolipoprotein A1, apoVLDII and the small heterodimer partner.

### A genome-wide scan reveals HNF4α's master function

**Figure 10:**
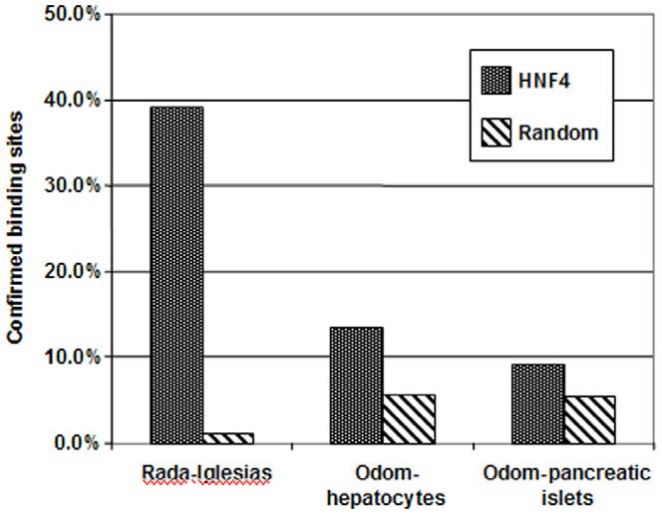
Comparison of HNF4α binding sites amongst different published studies. Percentage of HNF4α binding sites identified by Rada-Iglesias et al. [3] or Odom et al. [4] which could be confirmed in this study. For Rada-Iglesias et al. a control group of random genomic sequences was used to calculate the random overlap. For Odom et al., a control group was created by selecting randomly a number of promoter regions from the Huk13 array used in their study, equal to the number of promoters they detected as being bound by HNF4α.

Data from the present study was compared with published data in order to identify regions which overlap amongst these studies (**Fig. 10**). Of the 194 ES reported within the ENCODE regions [3], 76 overlapped with findings of the present study. Unfortunately, the ENCODE regions comprise 1% of the entire genome only. Furthermore, in a promoter-focused study [4] 1,553 bound sequences were reported for hepatocytes. In the present study and by selecting comparable sequence regions a total of 575 binding sites could be investigated. Of these, 200 binding sites were in common, therefore reconfirming 13% of the proposed promoter binding sites. Furthermore, the same investigator reported ES for pancreatic islets but only 9% could be confirmed in the present study with IP DNA from the Caco-2 cell line. Such differences may arise from the different experimental protocols and differences in cell types.

### Biological ontologies of *de novo* identified HNF4α gene targets

Based on Gene Ontology the *de novo* identified genes were grouped (**Table S9**). Many of the targeted genes are involved in different metabolic processes, e.g. lipid, organic acid or carbohydrate metabolism. Categories related to transport, i.e. lipid transport, were significantly overrepresented as was fatty acid and cholesterol metabolism [1], [22]. Additionally, many genes for development and differentiation were identified therefore reassuring HNF4α's role in development [23] and epithelial differentiation [22], [24]–[26]. This protein also controls the insulin secretory pathway [27] and is linked to rare monogenic disorder, i.e. maturity-onset diabetes of the young (MODY) [28]. Thus, genes targeted by HNF4α in the insulin signaling pathway as well as such related to cell death and tumour suppressor activity were identified [29].

### Defining functional binding sites - Correlation between genome-wide HNF4α ChIP-chip and gene expression data

**Figure 11:**
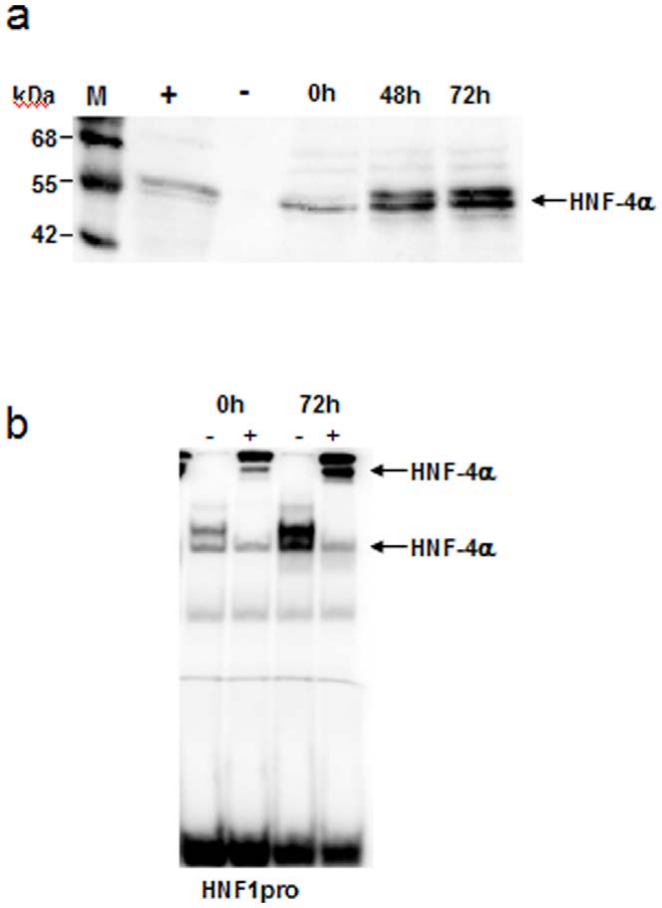
HNF4α protein expression and DNA binding activity with nuclear extracts isolated from Aroclor 1254 treated Caco-2 cells. a) HNF4α Western blotting of 20 µg Caco-2 cell extract. A clear induction of HNF4α protein expression was seen after 48 h and 72 h of Aroclor1254 treatment. b) Electrophoretic mobility shift assays with 2.5 µg Caco-2 cell nuclear extract and oligonucleotides corresponding to the A-site of the HNF1α promoter (HNF1pro) as 32P labeled probe. In supershift assays an antibody directed against HNF4α (+) was added. Binding of HNF4α was significantly increased after 72 h of Aroclor1254 induction.

A common approach to identify genes targeted by a transcription factor is to determine mRNA abundance caused by its increased or diminished transcriptional activity as investigated in human embryonic kidney (HEK293 [30]) and hepatoma cells (HUH7 [31], HepG2 [32]). Surprisingly, HNF4α transfection experiments influenced transcription of a small number of genes only. While it is known that transcriptional regulation is not mediated at the level of DNA binding alone [33] in such experiments most transcription factors bind under ‘non-activating’ conditions. To confirm functional binding sites of *de novo* identified HNF4α gene targets, Caco-2 cell cultures were treated with an inducer of HNF4α protein [34]. After treatment of Caco-2 cells with Aroclor 1254, binding of the HNF4α protein to the *HNF1α* promoter was increased [7] while the induction of the protein was confirmed by Western blotting experiments (**Fig. 11**). The Aroclor 1254 treated cultures were subjected to genome-wide transcript profiling. Using stringent criteria, 536 unique RefSeq-annotated genes were defined as differentially expressed (**Table S10**). Of these, 383 genes were up-regulated and 153 down regulated. The promoter sequences of regulated genes were analyzed for HNF4α binding sites and compared with the list of newly identified ChIP-chip gene targets. An overlap of 63% or 336 differentially expressed genes (**Table S11**) were identified as HNF4α gene targets, therefore confirming the functional importance of the ES identified in the ChIP-chip assay.

Finally, published data on HNF4α overexpressing mammalian cell lines was compared with data of the present study. The overlap ranged from 65 to 94% of the genes identified (**Table S10**). Importantly, the highest overlap was obtained in studies that employed knock-down siRNA experiments to validate their findings [32]. Therefore, gene targets reported here might be considered more reliable.

## Discussion

In the past, research on trans-acting factors and their corresponding *cis*-elements focused on promoter-proximal binding sites. With the development of ChIP-chip assays, genome-wide scans for transcription factor binding sites became feasible. This improved considerably an understanding of basic mechanisms of transcriptional control and an identification of promoter distal binding sites facilitating RNA processing events.

In the present study, a genome-wide map of HNF4α binding sites was constructed. This protein plays an essential role in liver development, and its master regulatory role in the maintenance of the metabolic competence of the liver has stimulated research on HNF4α targeted cancer therapies for its ability to revert liver cancer to a less aggressive phenotype [35].

The present study evidences >90% of the HNF4α binding sites to be located in promoter-distal regions and this distribution of ES is similar to that reported for the ERα [13]. Notably, with the exception of regions closer than 600 base pairs to the TSS, binding sites were more frequently downstream. Therefore, ChIP-chip assays focusing on promoter regions only [4], [36] might miss the majority of binding sites.

Moreover, an analysis of HNF4α motifs within ChIP-enriched regions demonstrates as high accuracy as in identification of HNF4α binding sites as achieved by the use of high resolution tiling array.

Importantly, an unexpected high number of HNF4α binding sites were identified. Even with stringent criteria, i.e. reproducible identification of enriched regions by three different algorithms, three independent ChIP experiments and strict exclusion of repetitive elements, >17,500 binding sites could be mapped.

Moreover, the distribution of identified ES across the chromosomes varied considerably and the chromosomal distribution of ES was not randomly distributed; rather clusters were identified (**Fig. 6a** **,** **Fig. 7**). Evidence was also obtained for the Y chromosome to be devoid of HNF4α ES (**Supplementary Table S6**). Note, of the 41 known and 14 novel protein coding genes on the Y chromosome (http://www.ensembl.org/index.html) several function in testis development and gender determination and include the SRY and TSPY proteins. While no vital genes are located on the Y chromosome several diseases can be linked to defective Y chromosome. It is tempting to speculate that the significant overlap between the ERα and the HNF4α motif and the functional interaction of these transcription factors possible determined, at least in part, evolution and thus gene selection of the Y chromosome.

Note, enhancer elements are constituted by clusters of binding sites for different transcription factors [37]. The increased conservation of the detected HNF4α binding sites, together with the highly significant enrichment of binding motifs of several other transcription factors in close vicinity, is suggestive for the identified binding sites to be functionally important and to possibly form an enhanceosome. Several sequence motifs were thus identified to be significantly overrepresented in ES regions of HNF4α while CART was significantly underrepresented as compared to the genomic background.

Based on a thorough and detailed motif analysis, a close relationship between HNF4α and AP1, GATA, ERα or HNF1α binding could be established. While single cases of synergistic action of HNF4α with HNF1α [38], ERα [39] or GATA transcription factors [32] had already been reported, their general importance is now demonstrated. To the best of our knowledge a cooperation of HNF4α with AP1 at HNF4α enriched binding sites has not been reported so far.

To further probe the functional importance of the newly identified binding sites transcript expression of targeted genes at varying HNF4α protein levels was studied. These studies confirmed 536 unique RefSeq-annotated genes to be differentially expressed. The promoter sequences of regulated genes were further analyzed and 63% or 336 differentially expressed genes (**Table S11**) were identified as HNF4α gene targets, therefore confirming the functional importance of the ES identified in the ChIP-chip assay. Hence, for some of the identified genes the biological relevance could be established. Nonetheless, further studies are in need to determine functional importance for the many other ES sites, and this could possibly be achieved with an additional control in ChIP-chip studies where the DNA binding domain of HNF4α has been disabled.

Additionally, over 90% of the ES are promoter distal sequences but could be defined as enhancer elements, while promoter proximal sites were also identified and compared to random controls. This suggests for HNF4α to interact directly and indirectly with the basal transcriptional machinery. The actual number of promoter-proximal binding sites will even be higher, as the TSS of RefSeq annotated genes were examined only. In the past, enhancers could hardly be identified by the available methods; with the advent of genomic platform technologies the comprehensive mapping of transcription factors is enabled to define enhancer elements, as attempted within the ENCODE regions [40].

Overall, HNF4α is truly a master regulatory protein in the orchestration of a wide range of biological processes. Knowledge on genes targeted by HNF4α will thus help to decipher the genetic basis of fundamental biological processes and its aberrant regulation in diseases [7], [41], [42].

In conclusion, a genome-wide map of HNF4α binding sites is reported to better understand basic mechanisms of transcriptional control of HNF4α targeted genes. Novel promoter distal binding sites are identified in facilitating RNA processing events and a gene repository is made available that is of utility in obtaining fundamental knowledge on the basic genetic events in disease.

## Materials and Methods

### Caco-2 Cell Culture, Chromatin immunoprecipitation (ChIP) and Aroclor 1254 treatment

Caco-2 cell culture, ChIP and chromatin preparation were performed as previously described [41], with the exception that the blocking steps with herring sperm DNA were omitted. High specificity of the antibody against HNF4α (Santa Cruz sc 6556×) used for the IP was confirmed by Western blot analysis. After ChIP, enrichment of two binding sites in the promoter regions of *HNF1α* and *AGT* was confirmed by quantitative real time PCR while Aroclor treatment was performed as described previously [34].

### ChIP-chip assay

Three samples displaying high enrichment for positive controls were selected for ChIP-chip experiments. Total input DNA from three independent biological replicates was diluted to the same concentration as the corresponding ChIP-sample and amplified in parallel with ChIP-samples from the three independent biological replicates. Amplification was performed according to the Affymetrix protocol. The cycle number and amount of *taq* polymerase was optimized for unbiased amplification. For fragmentation and labeling of the amplified DNA, the GeneChIP WT Double-Stranded DNA Terminal Labeling Kit from Affymetrix (P/N 900812) was used. Fragmentation success was confirmed with he Agilent Bioanalyzer 2100. The labeled samples were hybridized to Affymetrix Human tiling 2.0R arrays with a 35 base pair resolution.

Raw data (CEL-files generated by GCOS after scanning) were analyzed for enriched regions by three independent algorithms, TAS [8], MAT [9] and Tilemap [10]. The following parameters were chosen for ES identification: MAT bandwidth = 200, maximum gap = 300, minimum probes = 8, P-value<0.00001 and MAT score>5; Tilemap truncation = −1000000, 1000000, transform = none, GAP< = 300/probes between peaks< = 5, minimum length 200 nt / 5 probes, region summary method = HMM (a peak 28 probes on average, cutoff 0.5), FDR = left tail and FDR<0.015; TAS bandwidth = 400, P-value<0.01, minimum run = 200 and maximum gap = 250. Resulting regions were intersected using the Galaxy tool (http://g2.trac.bx.psu.edu/). After intersection enriched regions shorter than 200 bp were removed.

### ChIP and enrichment validation by real-time PCR

ChIP-DNA from three independent experiments was used for further validation. Realtime PCR was performed on the Light Cycler (Roche Diagnostics, Mannheim, Germany) with the following conditions: denaturation at 94°C for 120 s, extension at 72°C for different times and fluorescence at different temperatures. Primer sequences, annealing times and temperatures, extension times and fluorescence temperatures are summarized in **Table S12**. Δct-values were calculated versus diluted total input, and calculation of ΔΔct-values was performed using a β-actin negative control.

### Sequence conservation analysis

ES centers or peak positions detected by MAT were analyzed with CEAS [43] for conservation and motif content (see **Fig. 3**). For conservation analysis, CEAS extends genomic regions to 3,000 bp, and calculates for each nucleotide the average conservation score, based on the high-quality phast-Cons [44] information from the UCSC Genome Browser (http://genome.ucsc.edu/). The average conservation scores were plotted against the nucleotides position.

### Analysis of sequences for TF binding motifs

Sequence analysis for the detection of TF binding motifs was done with MATCH [11] and CisGenome (http://www.biostat.jhsph.edu/~hji/cisgenome/). Additionally, identification of enriched motifs within ChIP-chip detected regions was done with CEAS [43] and the Genomatix RegionMiner (http://www.genomatix.de/index.html).

### Correlation of HNF4α binding sites to RefSeq annotated genes and Gene Ontology categorization

The distribution of binding sites relative to TSS was analyzed, and a list of all RefSeq genes and their TSS was obtained from http://genome.ucsc.edu/. The closest TSS to each ES center was calculated in the application Microsoft Excel.

Enrichment of binding sites in introns was determined in RefSeq genes and their intron/exon structure was obtained from http://genome.ucsc.edu/. The number of ChIP regions and of regions from the random control set which overlap introns was determined using the intersect function of the Galaxy tool (http://g2.trac.bx.psu.edu/).

Association of binding sites identified by ChIP-chip with RefSeq annotated genes was performed with the software tool CisGenome (http://www.biostat.jhsph.edu/~hji/cisgenome/). ES were determined for all RefSeq genes with transcript coding regions within 100,000 bp from the center of the ES. All RefSeq genes associated with an ES were joined into a single list which was used for Gene Ontology categorization. Gene Ontology categorization was done with GOFFA [45].

### Expression profiling

Total RNA was isolated with the QIAGEN's RNeasy isolation kit and 10 µg of total RNA was used for subsequent hybridization experiments according to the manufacturer's recommendations and as described by Rohrbeck and Borlak [46]. Samples were hybridized to the Affymetrix U133Plus2.0 genechip arrays. The GCOS 1.4 software was used to calculate the level of differential expressed genes. Cutoff criteria for up- and down regulated genes were a logarithmized signal ratio >1.5, a present call and a signal >100 after 48 and 72 hours.

## Supporting Information

Table S1 Intersected_regions_and Peak Position.
(XLS)

Table S2 LowstringencySet.
(XLS)

Table S3 Motif enrichment analysis with MATCH [11]. Match analysis was performed with the ‘Vertebrate_all’ matrix set (578 matrices), with cutoff criteria set to minimize false positives. Regions analyzed were the 300 basepairs surrounding the peak positions. Motifs were counted and ratios between ChIP and random control regions were calculated. P-value was calculated based on a binomial distribution. To achieve stringent P-value calculations, the number of trials was set to “(region length - average motif length) * region number”. Cutoffs for enriched or depleted motifs were set to |Fold Change|>1,5 and P-value<1*10^−10^. Motifs with less than 50 hits in the HNF4α ChIP enriched regions and less than 25 hits in the random control regions have been excluded. P-Values<0E-15 were set to zero by Excel.
(DOC)

Table S4 Motif enrichment analysis with RegionMiner (Genomatix Software GmbH, Munich, Germany). Analysis of motif enrichment for single transcription factor matrices was performed with Genomatix RegionMiner. Cutoffs for enriched or depleted motifs were set to |Fold Change|>1,3 and |Z-score|>20.
(DOC)

Table S5 Enrichment analysis for transcription factor matrices families. Analysis of enrichment for transcription factor matrices families was performed with Genomatix RegionMiner. Cutoffs for enriched or depleted families were set to |Fold Change|>1,3 and |Z-score|>20.
(DOC)

Table S6 HNFα binding site frequency varies between chromosomes. The number of HNF4α binding sites on different chromosomes was compared with the number of RefSeq annotated genes and the length of the chromosomes. Chromosome length and gene numbers were retrieved from (http://genome.ucsc.edu/).
(DOC)

Table S7 HNF4α binding site clusters. HNF4α binding site clusters were identified by scanning the genome for regions containing 10 or more binding sites within a window of 100.000 bp. Genes with a TSS located within or close to these clusters with a high density of binding sites are given in the last column.
(DOC)

Table S8 Genes closest to novel HNF4alpha binding sites.
(XLS)

Table S9 Gene ontology terms of metabolism, development and transport are overrepresented among HNF4α RefSeq target genes identified in this study. Genes were analyzed with the Arraytrack Software tool GOFFA [45] for overrepresented ontologies. The 102 most significant terms fulfilling the cutoff criteria (P value<0.005; E value>1.2; hits ≥10) defining biological processes are given.
(DOC)

Table S10 Gene regulation upon Aroclor1254 treatment of Caco-2 cells.
(XLS)

Table S11 Comparison of RefSeq-annotated HNF4α targets identified by ChIP-chip to HNF4α targets identified by expression profiling in this study and in different publications. In the second column, the number of reported target genes from the relevant study, which could be associated to a current RefSeq annotation, is given. In the third column, the number of those RefSeq annotation is given, which could be also associated with a target gene identified in our ChIP-chip study. As 6670 from 18274 RefSeq Gene Symbols were identified as potential targets by ChIP-chip, the expected overlap by chance was 6670/18274*536 = 36%.
(DOC)

Table S12 Real-time PCR primer sequences and amplification protocol.
(DOC)
