# Supplementary material for: A High Resolution Genome-Wide Scan of HNF4α Recognition Sites Infers a Regulatory Gene Network in Colon Cancer
Source: PLoS One. 2011 Jul 28;6(7):e21667. doi: 10.1371/journal.pone.0021667 (PMC3145629; doi:10.1371/journal.pone.0021667)
Supplement: Table S3 — Motif enrichment analysis with MATCH [11]. Match analysis was performed with the ‘Vertebrate_all’ matrix set (578 matrices), with cutoff criteria set to minimize false positives. Regions analyzed were the 300 basepairs surrounding the peak positions. Motifs were counted and ratios between ChIP and random control regions were calculated. P-value was calculated based on a binomial distribution. To achieve stringent P-value calculations, the number of trials was set to “(region length - average motif length) * region number”. Cutoffs for enriched or depleted motifs were set to |Fold Change|>1,5 and P-value<1*10−10. Motifs with less than 50 hits in the HNF4α ChIP enriched regions and less than 25 hits in the random control regions have been excluded. P-Values<0E-15 were set to zero by Excel. (DOC) [file pone.0021667.s003.doc]

**Supplementary Table 3**

| **Motif ID** | **Motif Name** | **Hits**  **HNF4α** | **Hits Random**  **Control** | **Fold Change** | **P-Value** |
| --- | --- | --- | --- | --- | --- |
| M01031 | V$HNF4_Q6_01 | 2792 | 194 | 14.39 | 0.00E+00 |
| M00134 | V$HNF4_01 | 3108 | 231 | 13.45 | 0.00E+00 |
| M00411 | V$HNF4_01_B | 2947 | 296 | 9.96 | 0.00E+00 |
| M00764 | V$HNF4_DR1_Q3 | 2716 | 298 | 9.11 | 0.00E+00 |
| M00762 | V$DR1_Q3 | 2213 | 250 | 8.85 | 0.00E+00 |
| M00158 | V$COUP_01 | 3929 | 552 | 7.12 | 0.00E+00 |
| M00763 | V$PPAR_DR1_Q2 | 2122 | 367 | 5.78 | 0.00E+00 |
| M00765 | V$COUP_DR1_Q6 | 2390 | 452 | 5.29 | 0.00E+00 |
| M01036 | V$COUPTF_Q6 | 4103 | 794 | 5.17 | 0.00E+00 |
| M00528 | V$PPARG_03 | 1677 | 334 | 5.02 | 0.00E+00 |
| M00512 | V$PPARG_01 | 984 | 202 | 4.87 | 0.00E+00 |
| M00967 | V$HNF4_Q6 | 4512 | 1046 | 4.31 | 0.00E+00 |
| M00638 | V$HNF4ALPHA_Q6 | 4297 | 1028 | 4.18 | 0.00E+00 |
| M00080 | V$EVI1_03 | 94 | 33 | 2.85 | 0.00E+00 |
| M00082 | V$EVI1_05 | 94 | 33 | 2.85 | 0.00E+00 |
| M00242 | V$PPARA_01 | 7990 | 2974 | 2.69 | 0.00E+00 |
| M00959 | V$ER_Q6_02 | 645 | 258 | 2.50 | 0.00E+00 |
| M00462 | V$GATA6_01 | 82 | 34 | 2.41 | 1.37E-12 |
| M00495 | V$BACH1_01 | 356 | 152 | 2.34 | 0.00E+00 |
| M00926 | V$AP1_Q4_01 | 847 | 380 | 2.23 | 0.00E+00 |
| M00789 | V$GATA_Q6 | 3280 | 1473 | 2.23 | 0.00E+00 |
| M00790 | V$HNF1_Q6 | 1134 | 523 | 2.17 | 0.00E+00 |
| M00511 | V$ERR1_Q2 | 404 | 187 | 2.16 | 0.00E+00 |
| M00278 | V$LMO2COM_02 | 350 | 163 | 2.15 | 0.00E+00 |
| M00925 | V$AP1_Q6_01 | 1420 | 667 | 2.13 | 0.00E+00 |
| M01022 | V$LEF1_Q2_01 | 775 | 365 | 2.12 | 0.00E+00 |
| M00348 | V$GATA2_02 | 2117 | 998 | 2.12 | 0.00E+00 |
| M00736 | V$E2F1DP1_01 | 190 | 93 | 2.04 | 0.00E+00 |
| M00132 | V$HNF1_01 | 1245 | 610 | 2.04 | 0.00E+00 |
| M00174 | V$AP1_Q6 | 1782 | 905 | 1.97 | 0.00E+00 |
| M00671 | V$TCF4_Q5 | 2688 | 1382 | 1.95 | 0.00E+00 |
| M00206 | V$HNF1_C | 1449 | 750 | 1.93 | 0.00E+00 |
| M00517 | V$AP1_01 | 6056 | 3158 | 1.92 | 0.00E+00 |
| M00924 | V$AP1_Q2_01 | 1453 | 762 | 1.91 | 0.00E+00 |
| M00983 | V$MAF_Q6_01 | 2785 | 1462 | 1.90 | 0.00E+00 |
| M00427 | V$E2F_Q6 | 512 | 272 | 1.88 | 0.00E+00 |
| M00203 | V$GATA_C | 3416 | 1820 | 1.88 | 0.00E+00 |
| M00739 | V$E2F4DP2_01 | 292 | 156 | 1.87 | 0.00E+00 |
| M00123 | V$MYCMAX_02 | 220 | 119 | 1.85 | 0.00E+00 |
| M01132 | V$SF1_Q6_01 | 588 | 328 | 1.79 | 0.00E+00 |
| M00737 | V$E2F1DP2_01 | 216 | 121 | 1.79 | 2.14E-15 |
| M00260 | V$HLF_01 | 274 | 155 | 1.77 | 0.00E+00 |
| M00262 | V$STAF_01 | 189 | 107 | 1.77 | 2.38E-13 |
| M00199 | V$AP1_C | 6967 | 3956 | 1.76 | 0.00E+00 |
| M00978 | V$LEF1TCF1_Q4 | 2537 | 1443 | 1.76 | 0.00E+00 |
| M00428 | V$E2F1_Q3 | 209 | 119 | 1.76 | 2.55E-14 |
| M00188 | V$AP1_Q4 | 1612 | 922 | 1.75 | 0.00E+00 |
| M00025 | V$ELK1_02 | 1371 | 786 | 1.74 | 0.00E+00 |
| M00418 | V$TGIF_01 | 348 | 202 | 1.72 | 0.00E+00 |
| M00917 | V$CREB_Q4_01 | 441 | 256 | 1.72 | 0.00E+00 |
| M00807 | V$EGR_Q6 | 208 | 121 | 1.72 | 1.94E-13 |
| M00805 | V$LEF1_Q2 | 12639 | 7398 | 1.71 | 0.00E+00 |
| M00224 | V$STAT1_01 | 1181 | 696 | 1.70 | 0.00E+00 |
| M00173 | V$AP1_Q2 | 1314 | 775 | 1.70 | 0.00E+00 |
| M00128 | V$GATA1_04 | 2061 | 1221 | 1.69 | 0.00E+00 |
| M00538 | V$HTF_01 | 232 | 138 | 1.68 | 7.95E-14 |
| M00243 | V$EGR1_01 | 270 | 161 | 1.68 | 0.00E+00 |
| M00172 | V$AP1FJ_Q2 | 1125 | 671 | 1.68 | 0.00E+00 |
| M00916 | V$CREB_Q2_01 | 516 | 308 | 1.68 | 0.00E+00 |
| M01078 | V$CETS1P54_03 | 2134 | 1284 | 1.66 | 0.00E+00 |
| M00691 | V$ATF1_Q6 | 692 | 417 | 1.66 | 0.00E+00 |
| M00426 | V$E2F_Q4 | 984 | 594 | 1.66 | 0.00E+00 |
| M00292 | V$FREAC4_01 | 1469 | 899 | 1.63 | 0.00E+00 |
| M00187 | V$USF_Q6 | 602 | 369 | 1.63 | 0.00E+00 |
| M00179 | V$CREBP1_Q2 | 1458 | 894 | 1.63 | 0.00E+00 |
| M00225 | V$STAT3_01 | 715 | 441 | 1.62 | 0.00E+00 |
| M00981 | V$CREBATF_Q6 | 1924 | 1192 | 1.61 | 0.00E+00 |
| M00964 | V$PXR_Q2 | 1516 | 941 | 1.61 | 0.00E+00 |
| M00223 | V$STAT_01 | 969 | 603 | 1.61 | 0.00E+00 |
| M00178 | V$CREB_Q4 | 1328 | 829 | 1.60 | 0.00E+00 |
| M00039 | V$CREB_01 | 1520 | 949 | 1.60 | 0.00E+00 |
| M00177 | V$CREB_Q2 | 1155 | 726 | 1.59 | 0.00E+00 |
| M00327 | V$PAX3_B | 1903 | 1201 | 1.58 | 0.00E+00 |
| M00118 | V$MYCMAX_01 | 258 | 164 | 1.57 | 2.82E-12 |
| M00113 | V$CREB_02 | 478 | 304 | 1.57 | 0.00E+00 |
| M00117 | V$CEBPB_02 | 3335 | 2123 | 1.57 | 0.00E+00 |
| M00350 | V$GATA3_02 | 1934 | 1242 | 1.56 | 0.00E+00 |
| M00121 | V$USF_01 | 934 | 602 | 1.55 | 0.00E+00 |
| M01011 | V$HNF1_Q6_01 | 1704 | 1099 | 1.55 | 0.00E+00 |
| M00338 | V$ATF_B | 596 | 385 | 1.55 | 0.00E+00 |
| M00796 | V$USF_Q6_01 | 560 | 367 | 1.53 | 0.00E+00 |
| M00126 | V$GATA1_02 | 1852 | 1216 | 1.52 | 0.00E+00 |
| M00821 | V$NRF2_Q4 | 4653 | 3056 | 1.52 | 0.00E+00 |
| M00678 | V$TEL2_Q6 | 1198 | 788 | 1.52 | 0.00E+00 |
| M01070 | V$CMAF_01 | 1724 | 1136 | 1.52 | 0.00E+00 |
| M00245 | V$EGR3_01 | 440 | 290 | 1.52 | 0.00E+00 |
| M00119 | V$MAX_01 | 534 | 352 | 1.52 | 0.00E+00 |
| M00037 | V$NFE2_01 | 3744 | 2468 | 1.52 | 0.00E+00 |
| M00347 | V$GATA1_06 | 3479 | 2298 | 1.51 | 0.00E+00 |
| M00483 | V$ATF6_01 | 492 | 325 | 1.51 | 0.00E+00 |
| M00290 | V$FREAC2_01 | 2021 | 1336 | 1.51 | 0.00E+00 |
| M00017 | V$ATF_01 | 1427 | 945 | 1.51 | 0.00E+00 |
| M00339 | V$ETS1_B | 2755 | 1828 | 1.51 | 0.00E+00 |
| M00471 | V$TBP_01 | 3816 | 5815 | -1.52 | 0.00E+00 |
| M00101 | V$CDXA_02 | 4915 | 7569 | -1.54 | 0.00E+00 |
| M00342 | V$OCT1_B | 420 | 649 | -1.55 | 0.00E+00 |
| M00463 | V$POU3F2_01 | 7386 | 11423 | -1.55 | 0.00E+00 |
| M00630 | V$FOXM1_01 | 557 | 862 | -1.55 | 0.00E+00 |
| M00803 | V$E2F_Q2 | 647 | 1005 | -1.55 | 0.00E+00 |
| M00422 | V$FOXJ2_01 | 1426 | 2249 | -1.58 | 0.00E+00 |
| M00416 | V$CART1_01 | 1487 | 2361 | -1.59 | 0.00E+00 |
| M00930 | V$OCT1_Q5_01 | 420 | 671 | -1.60 | 0.00E+00 |
| M00991 | V$CDX_Q5 | 7526 | 12183 | -1.62 | 0.00E+00 |
| M00465 | V$POU6F1_01 | 196 | 319 | -1.63 | 3.14E-14 |
| M00639 | V$HNF6_Q6 | 348 | 634 | -1.82 | 0.00E+00 |
| M00414 | V$AREB6_03 | 422 | 826 | -1.96 | 0.00E+00 |
| M00510 | V$LHX3_01 | 89 | 181 | -2.03 | 1.28E-14 |
| M00220 | V$SREBP1_01 | 917 | 1952 | -2.13 | 0.00E+00 |
| M00233 | V$MEF2_04 | 422 | 1136 | -2.69 | 0.00E+00 |
| M00482 | V$PITX2_Q2 | 71 | 206 | -2.90 | 0.00E+00 |
