# Supplementary material for: A High Resolution Genome-Wide Scan of HNF4α Recognition Sites Infers a Regulatory Gene Network in Colon Cancer
Source: PLoS One. 2011 Jul 28;6(7):e21667. doi: 10.1371/journal.pone.0021667 (PMC3145629; doi:10.1371/journal.pone.0021667)
Supplement: Table S4 — Motif enrichment analysis with RegionMiner (Genomatix Software GmbH, Munich, Germany). Analysis of motif enrichment for single transcription factor matrices was performed with Genomatix RegionMiner. Cutoffs for enriched or depleted motifs were set to |Fold Change|>1,3 and |Z-score|>20. (DOC) [file pone.0021667.s004.doc]

**Supplementary Table S4**

| **TF Matrices** | **Matches** | **Expected** | **Std.dev.** | **Fold Change** | **Z-Score** | **Promoter association** |
| --- | --- | --- | --- | --- | --- | --- |
| V$HNF4.03 | 12066 | 3317 | 57.58 | 3.64 | 151.94 | No |
| V$HNF4.01 | 16287 | 4659 | 68.24 | 3.5 | 170.39 | no |
| V$HPF1.01 | 15940 | 4873 | 69.79 | 3.27 | 158.56 | no |
| V$COUP.01 | 9616 | 3242 | 56.93 | 2.97 | 111.97 | no |
| V$PPAR_RXR.02 | 12454 | 4277 | 65.39 | 2.91 | 125.04 | no |
| V$ARP1.01 | 9140 | 3270 | 57.17 | 2.8 | 102.66 | no |
| V$TR4.01 | 12105 | 4896 | 69.96 | 2.47 | 103.04 | no |
| V$PPARA.01 | 11176 | 5034 | 70.93 | 2.22 | 86.58 | no |
| V$LXRE.02 | 8034 | 3625 | 60.2 | 2.22 | 73.22 | no |
| V$RAR_RXR.01 | 8890 | 4009 | 63.3 | 2.22 | 77.1 | no |
| V$HNF4.02 | 6917 | 3166 | 56.26 | 2.18 | 66.66 | no |
| V$TR2_TR4.01 | 8859 | 4258 | 65.24 | 2.08 | 70.52 | no |
| V$PPAR_RXR.01 | 12625 | 6238 | 78.96 | 2.02 | 80.88 | no |
| V$NBRE.01 | 8210 | 4178 | 64.63 | 1.96 | 62.37 | no |
| V$RXR_RXR.01 | 4807 | 2482 | 49.82 | 1.94 | 46.65 | no |
| V$ERR.01 | 5931 | 3170 | 56.3 | 1.87 | 49.03 | no |
| V$TR4.02 | 8661 | 4750 | 68.9 | 1.82 | 56.76 | no |
| V$AP1.03 | 6269 | 3534 | 59.43 | 1.77 | 46.01 | no |
| V$COUP.02 | 3450 | 2010 | 44.83 | 1.72 | 32.1 | no |
| V$NFE2.01 | 4459 | 2823 | 53.13 | 1.58 | 30.78 | no |
| V$FTF.01 | 4905 | 3180 | 56.38 | 1.54 | 30.59 | no |
| V$LEF1.02 | 10997 | 7182 | 84.72 | 1.53 | 45.02 | no |
| V$RORA.01 | 5135 | 3405 | 58.34 | 1.51 | 29.65 | no |
| V$AP1.01 | 8529 | 5652 | 75.16 | 1.51 | 38.28 | no |
| V$MOK2.02 | 8126 | 5416 | 73.57 | 1.5 | 36.83 | no |
| V$ELK1.02 | 3021 | 2020 | 44.94 | 1.5 | 22.26 | yes |
| V$BACH2.01 | 5952 | 4047 | 63.6 | 1.47 | 29.94 | no |
| V$GKLF.02 | 7697 | 5346 | 73.1 | 1.44 | 32.15 | no |
| V$LEF1.01 | 12387 | 8665 | 93.05 | 1.43 | 39.99 | no |
| V$TR2.01 | 4577 | 3246 | 56.97 | 1.41 | 23.36 | no |
| V$DREAM.01 | 3767 | 2663 | 51.6 | 1.41 | 21.38 | no |
| V$RORA1.01 | 4384 | 3187 | 56.44 | 1.38 | 21.2 | no |
| V$PNR.01 | 8575 | 6222 | 78.85 | 1.38 | 29.84 | no |
| V$GATA1.01 | 5227 | 3806 | 61.68 | 1.37 | 23.03 | no |
| V$STAT1.01 | 7501 | 5510 | 74.21 | 1.36 | 26.82 | no |
| V$AP1.02 | 9626 | 7096 | 84.21 | 1.36 | 30.04 | no |
| V$MOK2.01 | 5138 | 3818 | 61.78 | 1.35 | 21.36 | no |
| V$ELK1.01 | 4596 | 3395 | 58.26 | 1.35 | 20.6 | yes |
| V$FXRE.01 | 4748 | 3549 | 59.56 | 1.34 | 20.13 | no |
| V$BACH1.01 | 6266 | 4683 | 68.41 | 1.34 | 23.14 | no |
| V$REV-ERBA.02 | 6720 | 5078 | 71.24 | 1.32 | 23.05 | no |
| V$STAT3.01 | 7331 | 5533 | 74.37 | 1.32 | 24.16 | no |
| V$GABP.01 | 6292 | 4765 | 69.01 | 1.32 | 22.13 | no |
| V$GATA1.06 | 6878 | 5231 | 72.3 | 1.31 | 22.78 | no |
| V$EN1.01 | 11072 | 14562 | 120.59 | -1.32 | -28.94 | no |
| V$XFD2.01 | 7286 | 9659 | 98.24 | -1.33 | -24.16 | no |
| V$GSH1.01 | 10644 | 14127 | 118.78 | -1.33 | -29.33 | no |
| V$CDX2.01 | 9664 | 12860 | 113.33 | -1.33 | -28.2 | no |
| V$CDX1.01 | 5538 | 7340 | 85.64 | -1.33 | -21.04 | no |
| V$OCT1.01 | 10984 | 14749 | 121.36 | -1.35 | -31.03 | no |
| V$NKX61.02 | 8488 | 11446 | 106.93 | -1.35 | -27.67 | no |
| V$BRN2.01 | 11391 | 15305 | 123.62 | -1.35 | -31.66 | no |
| V$BRN5.01 | 12157 | 16384 | 127.9 | -1.35 | -33.06 | no |
| V$CUT2.01 | 10445 | 14111 | 118.71 | -1.35 | -30.88 | no |
| V$SL1.01 | 8350 | 11436 | 106.88 | -1.37 | -28.88 | No |
| V$NKX61.01 | 14468 | 19802 | 140.59 | -1.37 | -37.94 | No |
| V$HHEX.01 | 11701 | 16188 | 127.14 | -1.39 | -35.3 | No |
| V$MEF2.01 | 7742 | 10922 | 104.45 | -1.41 | -30.45 | No |
| V$ATBF1.01 | 10950 | 15627 | 124.91 | -1.43 | -37.44 | No |
| V$OCT1P.01 | 11722 | 16718 | 129.19 | -1.43 | -38.67 | No |
| V$CLOX.01 | 8411 | 12149 | 110.16 | -1.45 | -33.94 | No |
| V$FHXB.01 | 11942 | 17541 | 132.33 | -1.47 | -42.32 | No |
| V$LHX3.01 | 13022 | 19082 | 138.01 | -1.47 | -43.91 | No |
| V$OCT1.06 | 12809 | 18939 | 137.49 | -1.47 | -44.59 | No |
| V$OC2.01 | 7139 | 10432 | 102.09 | -1.47 | -32.26 | No |
| V$BRN3.02 | 9073 | 13425 | 115.79 | -1.47 | -37.59 | No |
| V$LTATA.01 | 9774 | 14633 | 120.88 | -1.49 | -40.2 | No |
| V$MEF2.06 | 3396 | 5039 | 70.97 | -1.49 | -23.16 | No |
| V$BRN4.01 | 5197 | 7766 | 88.09 | -1.49 | -29.17 | No |
| V$BRN3.01 | 11330 | 17173 | 130.94 | -1.52 | -44.63 | No |
| V$MSX.01 | 8363 | 12650 | 112.41 | -1.52 | -38.15 | No |
| V$BRIGHT.01 | 7673 | 11560 | 107.46 | -1.52 | -36.18 | No |
| V$FREAC7.01 | 3934 | 6036 | 77.67 | -1.54 | -27.07 | No |
| V$XVENT2.01 | 5623 | 8663 | 93.04 | -1.54 | -32.68 | No |
| V$LHX3.02 | 12334 | 19280 | 138.72 | -1.56 | -50.07 | No |
| V$HFH3.01 | 1985 | 3219 | 56.73 | -1.61 | -21.76 | No |
| V$LMX1B.01 | 5910 | 9496 | 97.4 | -1.61 | -36.82 | No |
| V$CART1.01 | 5651 | 9152 | 95.62 | -1.61 | -36.62 | No |
| V$TATA.01 | 12400 | 21336 | 145.92 | -1.72 | -61.24 | No |
| V$BRN2.02 | 2871 | 5219 | 72.23 | -1.82 | -32.52 | No |
| V$BRN2.03 | 4148 | 7541 | 86.81 | -1.82 | -39.09 | No |
| V$SATB1.01 | 7527 | 14075 | 118.56 | -1.89 | -55.23 | No |
| V$PIT1.01 | 4222 | 8294 | 91.03 | -1.96 | -44.73 | No |
| V$CJUN_ATF2.01 | 594 | 1373 | 37.06 | -2.33 | -21.05 | No |
| V$MEF2.04 | 391 | 1175 | 34.27 | -3.03 | -22.88 | No |
| V$RAR_RXR.02 | 238 | 996 | 31.56 | -4.17 | -24.04 | No |
| V$CHOP.02 | 334 | 1521 | 39 | -4.55 | -30.46 | No |
