# Supplementary material for: A High Resolution Genome-Wide Scan of HNF4α Recognition Sites Infers a Regulatory Gene Network in Colon Cancer
Source: PLoS One. 2011 Jul 28;6(7):e21667. doi: 10.1371/journal.pone.0021667 (PMC3145629; doi:10.1371/journal.pone.0021667)
Supplement: Table S5 — Enrichment analysis for transcription factor matrices families. Analysis of enrichment for transcription factor matrices families was performed with Genomatix RegionMiner. Cutoffs for enriched or depleted families were set to |Fold Change|>1,3 and |Z-score|>20. (DOC) [file pone.0021667.s005.doc]

**Supplementary Table S5**

| **TF Families** | **Matches** | **Expected** | **Std. dev.** | **Fold Change** | **Z-Score** | **Promoter**  **association** |
| --- | --- | --- | --- | --- | --- | --- |
| V$PERO | 22293 | 10966 | 104.67 | 2.03 | 108.21 | No |
| V$NBRE | 8210 | 4178 | 64.63 | 1.96 | 62.37 | No |
| V$NR2F | 53199 | 30852 | 175.39 | 1.72 | 127.41 | no |
| V$EREF | 8903 | 5616 | 74.92 | 1.59 | 43.87 | no |
| V$LEFF | 19205 | 13266 | 115.11 | 1.45 | 51.59 | no |
| V$MOKF | 12274 | 8566 | 92.52 | 1.43 | 40.07 | no |
| V$CSEN | 3766 | 2663 | 51.6 | 1.41 | 21.36 | no |
| V$SF1F | 8556 | 6195 | 78.68 | 1.38 | 30 | no |
| V$RXRF | 44160 | 32677 | 180.49 | 1.35 | 63.62 | no |
| V$FXRE | 4748 | 3549 | 59.56 | 1.34 | 20.13 | no |
| V$AP1F | 14525 | 10858 | 104.15 | 1.34 | 35.2 | no |
| V$HOMF | 38771 | 51180 | 225.68 | -1.32 | -54.99 | no |
| V$NKX6 | 18696 | 24957 | 157.79 | -1.33 | -39.68 | no |
| V$CDXF | 13259 | 17748 | 133.11 | -1.33 | -33.73 | no |
| V$MEF2 | 18480 | 26465 | 162.47 | -1.43 | -49.15 | no |
| V$ATBF | 10949 | 15626 | 124.91 | -1.43 | -37.45 | no |
| V$OCTP | 11724 | 16718 | 129.19 | -1.43 | -38.66 | no |
| V$BRNF | 50781 | 74079 | 271.22 | -1.45 | -85.91 | no |
| V$LHXF | 22062 | 33608 | 183.03 | -1.52 | -63.08 | no |
| V$RBIT | 7672 | 11560 | 107.46 | -1.52 | -36.19 | no |
| V$CART | 10921 | 17137 | 130.8 | -1.56 | -47.53 | no |
| V$CHOP | 2053 | 3389 | 58.2 | -1.64 | -22.96 | no |
| V$SATB | 7526 | 14075 | 118.56 | -1.89 | -55.24 | no |
| V$PIT1 | 4222 | 8294 | 91.03 | -1.96 | -44.73 | no |
