# Supplementary material for: A High Resolution Genome-Wide Scan of HNF4α Recognition Sites Infers a Regulatory Gene Network in Colon Cancer
Source: PLoS One. 2011 Jul 28;6(7):e21667. doi: 10.1371/journal.pone.0021667 (PMC3145629; doi:10.1371/journal.pone.0021667)
Supplement: Table S6 — HNFα binding site frequency varies between chromosomes. The number of HNF4α binding sites on different chromosomes was compared with the number of RefSeq annotated genes and the length of the chromosomes. Chromosome length and gene numbers were retrieved from (http://genome.ucsc.edu/). (DOC) [file pone.0021667.s006.doc]

**Supplementary Table S6**

| **Chromosome** | **Genes** | **Total bases** | **Binding sites (BS)** | **BS / Gene** | **BS / 1.000.000 nucleotides** |
| --- | --- | --- | --- | --- | --- |
| chr1 | 2646 | 247249719 | 1827 | 0.69 | 0.74 |
| chr2 | 1584 | 242951149 | 1318 | 0.83 | 0.54 |
| chr3 | 1363 | 199501827 | 1150 | 0.84 | 0.58 |
| chr4 | 934 | 191273063 | 792 | 0.85 | 0.41 |
| chr5 | 1141 | 180857866 | 1074 | 0.94 | 0.59 |
| chr6 | 1286 | 170899992 | 1420 | 1.10 | 0.83 |
| chr7 | 1176 | 158821424 | 799 | 0.68 | 0.50 |
| chr8 | 896 | 146274826 | 695 | 0.78 | 0.48 |
| chr9 | 973 | 140273252 | 484 | 0.50 | 0.35 |
| chr10 | 1048 | 135374737 | 1137 | 1.08 | 0.84 |
| chr11 | 1608 | 134452384 | 982 | 0.61 | 0.73 |
| chr12 | 1296 | 132349534 | 978 | 0.75 | 0.74 |
| chr13 | 386 | 114142980 | 551 | 1.43 | 0.48 |
| chr14 | 810 | 106368585 | 629 | 0.78 | 0.59 |
| chr15 | 758 | 100338915 | 608 | 0.80 | 0.61 |
| chr16 | 1032 | 88827254 | 438 | 0.42 | 0.49 |
| chr17 | 1476 | 78774742 | 785 | 0.53 | 1.00 |
| chr18 | 353 | 76117153 | 353 | 1.00 | 0.46 |
| chr19 | 1677 | 63811651 | 212 | 0.13 | 0.33 |
| chr20 | 762 | 62435964 | 529 | 0.69 | 0.85 |
| chr21 | 329 | 46944323 | 146 | 0.44 | 0.31 |
| chr22 | 586 | 49691432 | 210 | 0.36 | 0.42 |
| chrX | 1315 | 154913754 | 432 | 0.33 | 0.28 |
| chrY | 134 | 57772954 | 6 | 0.04 | 0.01 |
| Total | 25569 | 3080419480 | 17561 | 0.69 | 0.57 |
