# Supplementary material for: A High Resolution Genome-Wide Scan of HNF4α Recognition Sites Infers a Regulatory Gene Network in Colon Cancer
Source: PLoS One. 2011 Jul 28;6(7):e21667. doi: 10.1371/journal.pone.0021667 (PMC3145629; doi:10.1371/journal.pone.0021667)
Supplement: Table S7 — HNF4α binding site clusters. HNF4α binding site clusters were identified by scanning the genome for regions containing 10 or more binding sites within a window of 100.000 bp. Genes with a TSS located within or close to these clusters with a high density of binding sites are given in the last column. (DOC) [file pone.0021667.s007.doc]

**Supplementary Table S7**

| **Chr** | **Start** | **Stop** | **Genes located within binding site cluster** |
| --- | --- | --- | --- |
| Chr1 | 116895000 | 117040000 | CD58, IGSF3 |
| Chr1 | 232725000 | 233080000 | IRF2BP2 |
| Chr3 | 125760000 | 125910000 | KALRN |
| Chr3 | 172150000 | 172300000 | SLC2A2, EIF5A2 |
| Chr3 | 195235000 | 195335000 | HES1 |
| Chr6 | 3270000 | 3405000 | SLC22A23 |
| Chr6 | 16185000 | 16310000 | MYLIP, GMPR |
| Chr6 | 167545000 | 167645000 | UNC93A, TTLL2 |
| Chr10 | 17135000 | 17295000 | CUBN, TRDMT1, VIM |
| Chr10 | 111660000 | 111820000 | XPNPEP1, ADD3 |
| Chr10 | 114045000 | 114550000 | TECTB, ACSL5, VTI1A, ZDHHC6 |
| Chr14 | 22325000 | 22445000 | SLC7A7**,** MRPL52, MMP14, LRP10, REM2 |
| Chr15 | 65120000 | 65220000 | SMAD3 |
| Chr17 | 46055000 | 46160000 | ABCC3, ANKRD40 |
| Chr17 | 67900000 | 68005000 | - |
