# Supplementary material for: A High Resolution Genome-Wide Scan of HNF4α Recognition Sites Infers a Regulatory Gene Network in Colon Cancer
Source: PLoS One. 2011 Jul 28;6(7):e21667. doi: 10.1371/journal.pone.0021667 (PMC3145629; doi:10.1371/journal.pone.0021667)
Supplement: Table S9 — Gene ontology terms of metabolism, development and transport are overrepresented among HNF4α RefSeq target genes identified in this study. Genes were analyzed with the Arraytrack Software tool GOFFA [45] for overrepresented ontologies. The 102 most significant terms fulfilling the cutoff criteria (P value<0.005; E value>1.2; hits ≥10) defining biological processes are given. (DOC) [file pone.0021667.s009.doc]

**Supplementary Table S9**

| **Term** | **GO ID** | **Level (Average)** | **P value (Average)** | **Gene Hits** | **E value** |
| --- | --- | --- | --- | --- | --- |
| intracellular signaling cascade | GO:0007242 | 5 | 0 | 469 | 1.24 |
| negative regulation of physiological process | GO:0043118 | 4 | 0 | 298 | 1.27 |
| negative regulation of cellular physiological process | GO:0051243 | 5 | 0 | 287 | 1.28 |
| negative regulation of cellular process | GO:0048523 | 4 | 0 | 318 | 1.28 |
| negative regulation of biological process | GO:0048519 | 3 | 0 | 343 | 1.28 |
| carboxylic acid metabolism | GO:0019752 | 6 | 0 | 203 | 1.4 |
| organic acid metabolism | GO:0006082 | 5 | 0 | 204 | 1.4 |
| lipid metabolism | GO:0006629 | 5 | 0 | 287 | 1.45 |
| cellular lipid metabolism | GO:0044255 | 5.25 | 0 | 231 | 1.47 |
| steroid metabolism | GO:0008202 | 6.25 | 0 | 82 | 1.64 |
| positive regulation of biological process | GO:0048518 | 3 | 0.000001 | 294 | 1.26 |
| phosphate metabolism | GO:0006796 | 6 | 0.000002 | 337 | 1.23 |
| phosphorus metabolism | GO:0006793 | 5 | 0.000002 | 337 | 1.23 |
| small GTPase mediated signal transduction | GO:0007264 | 6 | 0.000003 | 152 | 1.36 |
| regulation of signal transduction | GO:0009966 | 4.33 | 0.000005 | 150 | 1.35 |
| regulation of transferase activity | GO:0051338 | 4 | 0.000011 | 80 | 1.49 |
| regulation of kinase activity | GO:0043549 | 5 | 0.000013 | 79 | 1.49 |
| regulation of protein kinase activity | GO:0045859 | 6 | 0.000015 | 78 | 1.49 |
| protein amino acid dephosphorylation | GO:0006470 | 8 | 0.000015 | 67 | 1.54 |
| regulation of cell cycle | GO:0051726 | 5 | 0.000017 | 198 | 1.27 |
| lipid biosynthesis | GO:0008610 | 6 | 0.000018 | 103 | 1.41 |
| Dephosphorylation | GO:0016311 | 7 | 0.000022 | 72 | 1.5 |
| enzyme linked receptor protein signaling pathway | GO:0007167 | 6 | 0.000024 | 88 | 1.44 |
| positive regulation of cellular process | GO:0048522 | 4 | 0.000028 | 241 | 1.24 |
| negative regulation of metabolism | GO:0009892 | 5 | 0.000036 | 115 | 1.36 |
| regulation of progression through cell cycle | GO:0000074 | 6 | 0.000037 | 195 | 1.26 |
| transcription from RNA polymerase II promoter | GO:0006366 | 8 | 0.000039 | 199 | 1.26 |
| fatty acid metabolism | GO:0006631 | 6.57 | 0.000043 | 69 | 1.49 |
| alcohol metabolism | GO:0006066 | 5 | 0.000049 | 116 | 1.35 |
| protein amino acid phosphorylation | GO:0006468 | 8 | 0.00006 | 231 | 1.23 |
| positive regulation of physiological process | GO:0043119 | 4 | 0.000066 | 215 | 1.24 |
| regulation of small GTPase mediated signal transduction | GO:0051056 | 5.75 | 0.000082 | 61 | 1.5 |
| positive regulation of cellular physiological process | GO:0051242 | 5 | 0.000096 | 205 | 1.24 |
| amine metabolism | GO:0009308 | 5 | 0.000096 | 145 | 1.29 |
| cellular morphogenesis | GO:0000902 | 4.67 | 0.000109 | 118 | 1.33 |
| regulation of catalytic activity | GO:0050790 | 3 | 0.000112 | 123 | 1.32 |
| amino acid catabolism | GO:0009063 | 7.35 | 0.000118 | 31 | 1.76 |
| sensory perception of sound | GO:0007605 | 6 | 0.000139 | 54 | 1.52 |
| sensory perception of mechanical stimulus | GO:0050954 | 5 | 0.000139 | 54 | 1.52 |
| neurite development | GO:0031175 | 6.6 | 0.000143 | 34 | 1.71 |
| regulation of body fluids | GO:0050878 | 4 | 0.000156 | 53 | 1.52 |
| nitrogen compound catabolism | GO:0044270 | 5.8 | 0.000158 | 33 | 1.71 |
| neurite morphogenesis | GO:0048812 | 7.53 | 0.000174 | 32 | 1.72 |
| neuron morphogenesis during differentiation | GO:0048667 | 6.5 | 0.000174 | 32 | 1.72 |
| amine catabolism | GO:0009310 | 6.44 | 0.000174 | 32 | 1.72 |
| sterol metabolism | GO:0016125 | 6.71 | 0.000175 | 40 | 1.62 |
| Morphogenesis | GO:0009653 | 3 | 0.000179 | 242 | 1.21 |
| actin filament-based process | GO:0030029 | 7 | 0.000188 | 77 | 1.41 |
| nitrogen compound metabolism | GO:0006807 | 4 | 0.000189 | 153 | 1.27 |
| neuron development | GO:0048666 | 5.6 | 0.000204 | 42 | 1.59 |
| negative regulation of protein kinase activity | GO:0006469 | 6.25 | 0.00021 | 23 | 1.89 |
| negative regulation of transferase activity | GO:0051348 | 5 | 0.00021 | 23 | 1.89 |
| negative regulation of cellular metabolism | GO:0031324 | 6 | 0.000223 | 97 | 1.35 |
| intercellular junction assembly | GO:0007043 | 7 | 0.000242 | 11 | 2.51 |
| cellular morphogenesis during differentiation | GO:0000904 | 5.4 | 0.000249 | 33 | 1.68 |
| Coagulation | GO:0050817 | 3 | 0.00026 | 46 | 1.55 |
| negative regulation of transcription | GO:0016481 | 8 | 0.000303 | 81 | 1.38 |
| aromatic compound catabolism | GO:0019439 | 6 | 0.000326 | 12 | 2.37 |
| negative regulation of nucleobase, nucleoside, nucleotide and nucleic acid metabolism | GO:0045934 | 7 | 0.000371 | 85 | 1.36 |
| regulation of cell motility | GO:0051270 | 5.14 | 0.000429 | 21 | 1.88 |
| regulation of locomotion | GO:0040012 | 4 | 0.000429 | 21 | 1.88 |
| neuron differentiation | GO:0030182 | 5 | 0.000445 | 47 | 1.51 |
| Apoptosis | GO:0006915 | 6 | 0.000474 | 229 | 1.2 |
| actin filament depolymerization | GO:0030042 | 8 | 0.000487 | 16 | 2.06 |
| positive regulation of nucleobase, nucleoside, nucleotide and nucleic acid metabolism | GO:0045935 | 7 | 0.000534 | 64 | 1.41 |
| actin cytoskeleton organization and biogenesis | GO:0030036 | 8 | 0.000545 | 70 | 1.39 |
| system development | GO:0048731 | 3 | 0.000579 | 191 | 1.21 |
| membrane lipid biosynthesis | GO:0046467 | 7.11 | 0.000601 | 39 | 1.56 |
| blood coagulation | GO:0007596 | 5.5 | 0.000641 | 44 | 1.51 |
| regulation of cyclin-dependent protein kinase activity | GO:0000079 | 7 | 0.000653 | 23 | 1.79 |
| lipid transport | GO:0006869 | 5.33 | 0.000673 | 35 | 1.59 |
| cellular catabolism | GO:0044248 | 5 | 0.000675 | 195 | 1.21 |
| regulation of actin polymerization and/or depolymerisation | GO:0008064 | 7.05 | 0.000709 | 22 | 1.81 |
| protein kinase cascade | GO:0007243 | 6 | 0.000719 | 122 | 1.27 |
| positive regulation of transcription | GO:0045941 | 8 | 0.000764 | 61 | 1.41 |
| regulation of Rho protein signal transduction | GO:0035023 | 7 | 0.000764 | 37 | 1.56 |
| response to drug | GO:0042493 | 5 | 0.000822 | 20 | 1.85 |
| steroid biosynthesis | GO:0006694 | 7.11 | 0.000862 | 36 | 1.57 |
| amino acid and derivative metabolism | GO:0006519 | 5 | 0.000895 | 115 | 1.28 |
| intercellular junction assembly and maintenance | GO:0045216 | 6 | 0.000902 | 12 | 2.22 |
| Hemostasis | GO:0007599 | 5 | 0.000928 | 46 | 1.48 |
| regulation of cell migration | GO:0030334 | 6.21 | 0.000928 | 18 | 1.9 |
| nervous system development | GO:0007399 | 4 | 0.000961 | 188 | 1.2 |
| cholesterol metabolism | GO:0008203 | 7.71 | 0.000972 | 35 | 1.57 |
| generation of neurons | GO:0048699 | 6 | 0.001288 | 48 | 1.45 |
| wound healing | GO:0042060 | 5 | 0.001455 | 47 | 1.45 |
| cell migration | GO:0016477 | 5.4 | 0.001496 | 49 | 1.44 |
| sterol biosynthesis | GO:0016126 | 7.94 | 0.001677 | 18 | 1.84 |
| Neurogenesis | GO:0022008 | 5 | 0.00169 | 48 | 1.44 |
| negative regulation of progression through cell cycle | GO:0045786 | 6.4 | 0.001738 | 75 | 1.33 |
| membrane lipid metabolism | GO:0006643 | 6.25 | 0.001919 | 65 | 1.36 |
| positive regulation of cellular metabolism | GO:0031325 | 6 | 0.002 | 79 | 1.31 |
| positive regulation of metabolism | GO:0009893 | 5 | 0.002185 | 85 | 1.3 |
| amino acid metabolism | GO:0006520 | 6.27 | 0.002716 | 96 | 1.27 |
| phospholipid metabolism | GO:0006644 | 7.25 | 0.002834 | 48 | 1.41 |
| cell development | GO:0048468 | 4 | 0.00317 | 55 | 1.37 |
| Digestion | GO:0007586 | 4 | 0.003587 | 30 | 1.53 |
| transmembrane receptor protein serine/threonine kinase signaling pathway | GO:0007178 | 7 | 0.004099 | 23 | 1.62 |
| insulin receptor signaling pathway | GO:0008286 | 8 | 0.004223 | 13 | 1.92 |
| negative regulation of cell organization and biogenesis | GO:0051129 | 6 | 0.004232 | 19 | 1.7 |
| transmembrane receptor protein tyrosine kinase signaling pathway | GO:0007169 | 7 | 0.004284 | 58 | 1.34 |
| aspartate family amino acid metabolism | GO:0009066 | 7.27 | 0.004415 | 12 | 1.97 |
