# Supplementary material for: A High Resolution Genome-Wide Scan of HNF4α Recognition Sites Infers a Regulatory Gene Network in Colon Cancer
Source: PLoS One. 2011 Jul 28;6(7):e21667. doi: 10.1371/journal.pone.0021667 (PMC3145629; doi:10.1371/journal.pone.0021667)
Supplement: Table S11 — Comparison of RefSeq-annotated HNF4α targets identified by ChIP-chip to HNF4α targets identified by expression profiling in this study and in different publications. In the second column, the number of reported target genes from the relevant study, which could be associated to a current RefSeq annotation, is given. In the third column, the number of those RefSeq annotation is given, which could be also associated with a target gene identified in our ChIP-chip study. As 6670 from 18274 RefSeq Gene Symbols were identified as potential targets by ChIP-chip, the expected overlap by chance was 6670/18274*536 = 36%. (DOC) [file pone.0021667.s011.doc]

**Supplementary Table S11**

|  | **RefSeq-annotated HNF4α targets identified by expression profiling** | **Of these:**  **Also identified by ChIP-chip** | **% overlap** |
| --- | --- | --- | --- |
| **Aroclor treatment** | **536** | **336** | **63%** |
| **Sumi et al.** [32] | **39** | **37** | **95%** |
| **Naiki et al.** [31] | **60** | **38** | **63%** |
| **Lucas et al.** [30] | **56** | **39** | **70%** |
| **random overlap** |  |  | **36,5%** |
