# Supplementary material for: A High Resolution Genome-Wide Scan of HNF4α Recognition Sites Infers a Regulatory Gene Network in Colon Cancer
Source: PLoS One. 2011 Jul 28;6(7):e21667. doi: 10.1371/journal.pone.0021667 (PMC3145629; doi:10.1371/journal.pone.0021667)
Supplement: Table S12 — Real-time PCR primer sequences and amplification protocol. (DOC) [file pone.0021667.s012.doc]

**Supplementary Table S12**

| **Primer Name** | **Primer Sequence** | **Product**  **Length** | **Annealing** | **Extension** | **Fluorescence** |
| --- | --- | --- | --- | --- | --- |
| Val5-for | CCCTGAAGTGGACTGAGAGC | 151 | 68° 7´´ | 5´´ | 82° |
| Val5-rev | GCCACGAGGTGACAAGCTAT |  |  |  |  |
| Val6-for | AACCATCACTTGTTCTTTCATCC | 211 | 68° 7´´ | 5´´ | 78° |
| Val6-rev | TGAAGCCAAGGGTTGTATCA |  |  |  |  |
| Val7-for | CAATCTTCTGGCCCCATCTA | 252 | 68° 7´´ | 6´´ | 81° |
| Val7-rev | GCATGTGCCTGCATTACTGT |  |  |  |  |
| Val8-for | GATCACCGTGAGGCTAAGGA | 183 | 68° 7´´ | 5´´ | 81° |
| Val8-rev | AAGCTTGGCGTAGCTTTTGA |  |  |  |  |
| Val10-for | CTTTGGGGGAGGTGGATATT | 205 | 68° 7´´ | 5´´ | 81° |
| Val10-rev | CCCATGGGAAAAGCACTCTA |  |  |  |  |
| Val11-for | AAGTGCCGTGACCTGAAAGT | 202 | 68° 7´´ | 5´´ | 85° |
| Val11-rev | CCTGGCCACTGTGGTTATTA |  |  |  |  |
| Val12-for | GCGGTGAGGATGGTTACAGT | 220 | 69° 7´´ | 6´´ | 82° |
| Val12-rev | TTGCCAAGGGTCTGAAAATC |  |  |  |  |
| Val13-for | CCAGCTCCATACCTCTGCTC | 246 | 68° 7´´ | 6´´ | 82° |
| Val13-rev | GCAGCAGTTTGAGGCATTCT |  |  |  |  |
| Val14-for | GGAAATGACCATGCACATCA | 266 | 68° 7´´ | 7´´ | 85° |
| Val14-rev | CAGAGATCTCACAGGCCACA |  |  |  |  |
| Val9-for | ATGGAGTTTCTGCCATCCAC | 165 | 68° 7´´ | 5´´ | 82° |
| Val9-rev | TGTCAGGGTTTGGCTTTAGG |  |  |  |  |
| Val4-for | gccagtctctgacagggttc | 178 | 68° 8´´ | 7´´ | 86° |
| Val4-rev | gtccaggtgccacatctacc |  |  |  |  |
| Val3-for | cagtgctggccagagttaca | 151 | 68° 8´´ | 8´´ | 83° |
| Val3-rev | ctgggaatgcaaatccagtt |  |  |  |  |
| Val2-for | ctgtggaagcctggtaggtc | 177 | 68° 8´´ | 9´´ | 88° |
| Val2-rev | gccaaatggaactcaaggaa |  |  |  |  |
| Val15-for | taacgcgaatgaagcaacag | 144 | 68° 8´´ | 7´´ | 80° |
| Val15-rev | tggtcgacgagtgaactttg |  |  |  |  |
| Val1-for | aagtcactggaggagctgga | 195 | 68° 7´´ | 7´´ | 83° |
| Val1-rev | ctccaaagggcatggagata |  |  |  |  |
| ßACT-ChIP-for | CTCCTGAGCGCAAGTACTCC | 188 | 67° 8´´ | 9´´ | 87° |
| ßACT-ChIP-rev | GCGCAAGTTAGGTTTTGTCA |  |  |  |  |
| HNF1-ChIPupstream-for | CCTAAAGAAACCCAGCACCA | 213 | 68° 8´´ | 9´´ | 84° |
| HNF1-ChIPupstream-rev | GTTCCAGTACATGCCACAACA |  |  |  |  |
